# Prediction models for adverse events from prostate cancer curative radiotherapy: a systematic review of methodological quality

**DOI:** 10.1016/j.tranon.2026.102961

**Published:** 2026-08-08

**Authors:** Claudia Cruz Oliveira, Christian A M Jongen, Ana Mikolić, Luca Incrocci, Hester F Lingsma, Monique J Roobol, Ida Korfage, Wilma D Heemsbergen, David van Klaveren, on behalf of the 4D PICTURE consortium, Carlos Romero, Liesbeth C. de Wreede, Frank Doornkamp, Yassin Engelberts, Olesja Fornara, Kasper Frank, Toby Hackmann, Lára R. Hallsson, Lennart Koch, Daisy Monika Lal, Amara Callistus Nwosu, Roberto Pazo-Cid, Judith Rietjens, Uwe Siebert, E.W. Steyerberg, Elfi M. Verheul, Tamara Wit, Michel W. Wouters

**Affiliations:** d Fractal Strategy, Zaragoza, Spain; e Medical Statistics, Department of Biomedical Data Sciences, Leiden University Medical Center, Leiden, The Netherlands; f Department of Public Health, Erasmus MC, University Medical Center Rotterdam, Rotterdam, The Netherlands; g Department of Oncology-Pathology, Karolinska Institute, Stockholm, Sweden; h Center for Shared Decision Making, Vejle/Lillebaelt University Hospital of Southern Denmark, Denmark; i Institute of Public Health, Medical Decision Making and Health Technology Assessment; Department of Public Health, Health Services Research and Health Technology Assessment, UMIT TIROL – University for Health Sciences and Technology, Hall in Tirol, Austria; j School of Computing and Communication, Lancaster University, Lancaster, United Kingdom; k International Observatory on End of Life Care, Lancaster University, Lancaster, United Kingdom; Marie Curie North West, Liverpool, United Kingdom; l Department of Medical Oncology, Miguel Servet University Hospital; Facultad de Medicina, Universidad de Zaragoza; Instituto de Investigación Sanitaria Aragón (IIS-A), Zaragoza, Spain; m Department of Design, Organisation and Strategy, Faculty of Industrial Design Engineering, Delft University of Technology, Delft, The Netherlands; n Center for Health Decision Science, Departments of Epidemiology and Health Policy & Management, Harvard T.H. Chan School of Public Health, Boston, MA, USA; o Institute for Technology Assessment, Department of Radiology, Massachusetts General Hospital, Harvard Medical School, Boston, MA, USA; p Julius Center, University Medical Center Utrecht, Utrecht, The Netherlands; q Medical Decision Making, Department of Biomedical Data Sciences, Leiden University Medical Center, Leiden, The Netherlands; a Department of Public Health, Erasmus MC University Medical Center, Dr. Molewaterplein 40, 3015 GD Rotterdam, Rotterdam, The Netherlands; b Department of Radiotherapy, Erasmus MC Cancer Institute, Erasmus MC University Medical Center, Rotterdam, The Netherlands; c Department of Urology, Erasmus MC Cancer Institute, University Medical Center Rotterdam, Rotterdam, The Netherlands

**Keywords:** Prostate cancer, Radiotherapy, Complications, Radiation injuries, NTCP, Statistical models, Machine learning

## Abstract

**Background:**

Predicting risks of urinary, bowel, sexual, and other adverse effects following prostate cancer curative radiotherapy (PCa-RT) is essential for treatment personalization and patient counseling. Several clinical prediction models (CPMs) have been published; however, their methodological quality remains unclear.

**Methods:**

We systematically reviewed studies developing or validating CPMs for adverse events after PCa-RT. Embase and Medline were searched for CPM studies for patient- or clinician-reported outcomes (PROs/ClinROs), published between January 1, 2004, and August 6, 2024. To focus the appraisal on methodologically more robust models, models were pre-selected based on events per variable ≥10 or the reporting of optimism-corrected performance metrics or effect estimates. We appraised models based on performance and ROB, using a six-item short form of the Prediction model Risk Of Bias ASsessment Tool (SF-PROBAST).

**Results:**

Of 3606 records screened, 136 CPM studies were identified, and 35 were included, yielding 107 CPMs. Most models (*n* = 87) were developed in external beam radiation therapy populations. 22 models were externally validated. Only two models (AUC= 0.59 and 0.80) – developed in two different studies – were classified as low risk of bias (fulfilled all the SF-PROBAST criteria). 32 models, from 13 studies, met at least four SF-PROBAST criteria and showed at least moderate discrimination (AUC ≥ 0.70) at internal (*n* = 25) and/or external (*n* = 11) validation.

**Conclusion:**

Ready-to-use CPMs in PCa-RT remain scarce due to methodological limitations, miscalibration, and lack of external validation. Future efforts should prioritize validation and refinement of existing models rather than development of new ones.

## Highlights

• Review of 20 years of prediction models for adverse events after PCa-RT.
• Overview of performance, risk of bias, methods, and key predictors.
• No ready-to-use models identified, but 32 showed good performance and sound methods.
• Future work should focus on validation and refinement of existing models.

## Background

Radiotherapy is one of the main curative treatment options for localized prostate cancer [1]. Prostate cancer curative radiotherapy (PCa-RT) techniques have been continuously improved over the years to reduce the radiation delivered to healthy tissues, thereby reducing the incidence and severity of adverse events (AEs) [2]. However, despite these significant advances, PCa-RT treatments are still associated with urinary and bowel AEs (e.g., incontinence and voiding disorders, rectal bleeding, and persistent diarrhea), and sexual dysfunction (e.g., erectile dysfunction), among other AEs [[3], [4], [5], [6], [7]].

Clinical prediction models (CPMs) for AEs among patients undergoing PCa-RT – including pre-treatment models and Normal Tissue Complication Probability (NTCP) models – have several applications in clinical practice. Accounting for relevant baseline predictors such as patient characteristics (e.g., comorbidities, health status, medication use, prostate volume) and tumor characteristics (e.g., pathological and clinical staging) has been shown to enhance the predictive performance of these models and to enable individualized risk estimation [[8], [9], [10], [11]]. NTCP models that include baseline predictors are an example of CPMs that may be used to support personalized treatment planning [[12], [13], [14]]. This is possible since current radiotherapy (RT) techniques allow for precision treatments that tailor dose distributions to individual patients [[15], [16], [17]]. Additionally, CPMs that predict patient-reported outcomes (PROs) may also be used to manage patients’ expectations and guide supportive care planning for potential AEs [[18], [19], [20]]. Despite the value of individual risk predictions of AEs in PCa-RT patients, the implementation of CPMs in clinical practice is currently limited [21,22]. CPMs can be promising for clinical implementation if they provide additional and relevant information to current clinical knowledge, have a low risk of bias, and have a good predictive ability that has been confirmed in external datasets [23,24]. Previous systematic reviews of prediction models for different clinical fields have shown a high risk of bias for most published models according to the Prediction model Risk Of Bias ASsessment Tool (PROBAST), questioning the validity of the predictions provided [[25], [26], [27]]. Since a methodological appraisal of CPMs in the field of AEs in PCa-RT is currently lacking, the quality of these models is unclear, which may hamper their readiness for clinical practice. An overview of existing models and their quality assessment is crucial to elucidate the current state of the field, and may uncover gaps and highlight promising CPMs.

We aimed to systematically review studies that have developed or validated clinical prediction models, including baseline predictors of acute and late adverse events from prostate cancer curative treatment. Additionally, we aimed to critically appraise and identify the most promising models in the PCa-RT field based on performance and methodological soundness.

## Methods

This systematic literature review was performed according to the Transparent Reporting of a multivariable prediction model for Individual Prognosis Or Diagnosis – Systematic Reviews and Meta-Analysis (TRIPOD-SRMA; checklist in Supplementary A) [28]. We registered the protocol of this review in the International Prospective Register of Systematic Reviews (PROSPERO) database [29], under the ID number CRD42025617010.

### Search strategy and data sources

A medical librarian from the Erasmus MC medical library was involved in the development of the search strategy (Supplementary A). We performed the search in Embase and Medline databases.

### Eligibility criteria

#### Inclusion criteria

We included studies published from 1 January 2004 to 6 August 2024. This timeframe was chosen considering that RT techniques have been updated over time, and AEs patterns related to these treatments before 2004 are believed not to translate to current practice. We included prediction modeling studies, including external validation studies, that used baseline predictors such as patient- (e.g., age, comorbidities, medication intake) or tumor-related (e.g., clinical staging, baseline PSA, Gleason grade) characteristics to predict AEs measured using Patient-Reported Outcomes (PROs) or Clinician-Reported Outcomes (ClinROs), or other AEs related to PCa-RT (e.g., the need for medical interventions).

#### Exclusion criteria

We excluded studies if:

1. It was focused on recurrent prostate cancer patients.
2. It did not aim to develop a prediction model and did not provide information on model development strategies or performance measures (e.g., causal inference or descriptive studies, literature reviews, studies focused on finding predictors but without developing a multivariate prediction model)
3. It focused on outcome trajectories, such as longitudinal studies.
4. It did not include at least one baseline predictor in its final model.
5. It did not report the final model(s) in the form of a model equation, tool (e.g., nomogram, web-based tool), trained model, or the code to train a model with respective data.
6. It included model(s) or externally validated model(s) considered at high risk of optimism/overfitting (i.e., event per variable ratio < 10 [30] with no correction for model optimism through penalization techniques or corrected performance estimates). This criterion was added to preselect higher-quality models, since overfitted models may work well on development data but perform poorly in independent populations [31,32].

### Data screening and extraction

The data screening and extraction were performed independently by two researchers (CCO and CJ) after completing a shared screening phase. The first screening round —titles and abstracts— was shared for the 300 most recent studies (agreement= 89%). The second screening round —full-text screening— was shared for the 20 most recent studies identified in the previous round (agreement = 85%). Considering the high inter-rater agreement rate, the screening of the remaining studies was split equally between the two researchers.

Data extraction was performed as recommended by the CHecklist for critical Appraisal and data extraction for systematic Reviews of prediction Modelling Studies (CHARMS) (Supplementary B) [33]. To ensure consistency in the data extraction, discrepancies were resolved through discussion or, if needed, with input from co-authors (WH and DK), until consensus was reached. In agreement with the CHARMS guidelines, we extracted data on the study design, study population (including type(s) of RT administered), candidate and final model predictors, model development and validation methods, and performance measures of the included models.

We used Rayyan [34] for the screening of studies and performed data extraction with Microsoft Excel 2016.

### Data synthesis

The data synthesis depicted, in figures, the outcomes and the discriminative abilities (e.g., C‑statistic or area under the receiver operating characteristic curve– AUC) of identified models at development and external validation, and the calibration slopes of different models at external validation. Discrimination refers to a model's ability to distinguish between individuals at higher versus lower risk of an event. A discrimination value of 0.5 indicates no discriminative ability—equivalent to random chance—whereas a value of 1.0 represents perfect discrimination. The calibration slope assesses whether the strength of the predictors—reflected in the model's coefficients—is accurately estimated in the validation cohort. A calibration slope of 1 indicates perfect calibration; values <1 suggest that the predictor effects are overestimated, while values greater than 1 indicate underestimation.

For models with an acceptable methodological soundness (not failing to meet more than two criteria of the risk of bias assessment) and at least moderate performance at internal and if applicable, external validation (AUC ≥ 0.7), study characteristics —including study population(s), data source(s), outcome definition, final predictors, model development methods, and performance measures (e.g., AUC, Brier score or R^2^) at both development and validation —were synthesized in a summary table. The frequency of testing of baseline predictors and how often they were selected in final models were also synthesized in tables for the most common outcomes (Supplementary A).

The data synthesis was managed with Microsoft Excel 2016 (tables) and with the ggplot2 package R version 4.3.2 (figures).

### Risk of bias assessment

We used a previously proposed tool to assess the quality of the methodological approaches used for developing the included models (supplementary A) [35]. This tool — SF-PROBAST — consists of a short form including six out of 20 questions from the tool to Assess the Risk of Bias and Applicability of Prediction Model Studies (PROBAST) [30] and has been shown to effectively evaluate the risk of bias of prediction modeling studies [33]. It comprises the most relevant sources of bias that influence the quality of prediction models. The six questions reflect two PROBAST domains (Outcome and analysis), namely whether the same outcome definition was used for all subjects of the sampled population (Outcome assessment), if models were developed in large enough samples (Events per variable- EPV), if dichotomization of continuous predictors was avoided (Continuous predictors), if missing data for >5% of subjects was handled accordingly using multiple imputation (Missing data), if univariable analysis as a predictor selection method was avoided (Univariable analysis), and whether the performance of the models was corrected for optimism (Correction for overfitting/optimism). When using the SF-PROBAST, models can be classified as either low-risk of bias (no risk of bias identified) or as high-risk of bias (fail to meet at least one of the criteria).

## Results

### Literature search

**Fig. 1:**
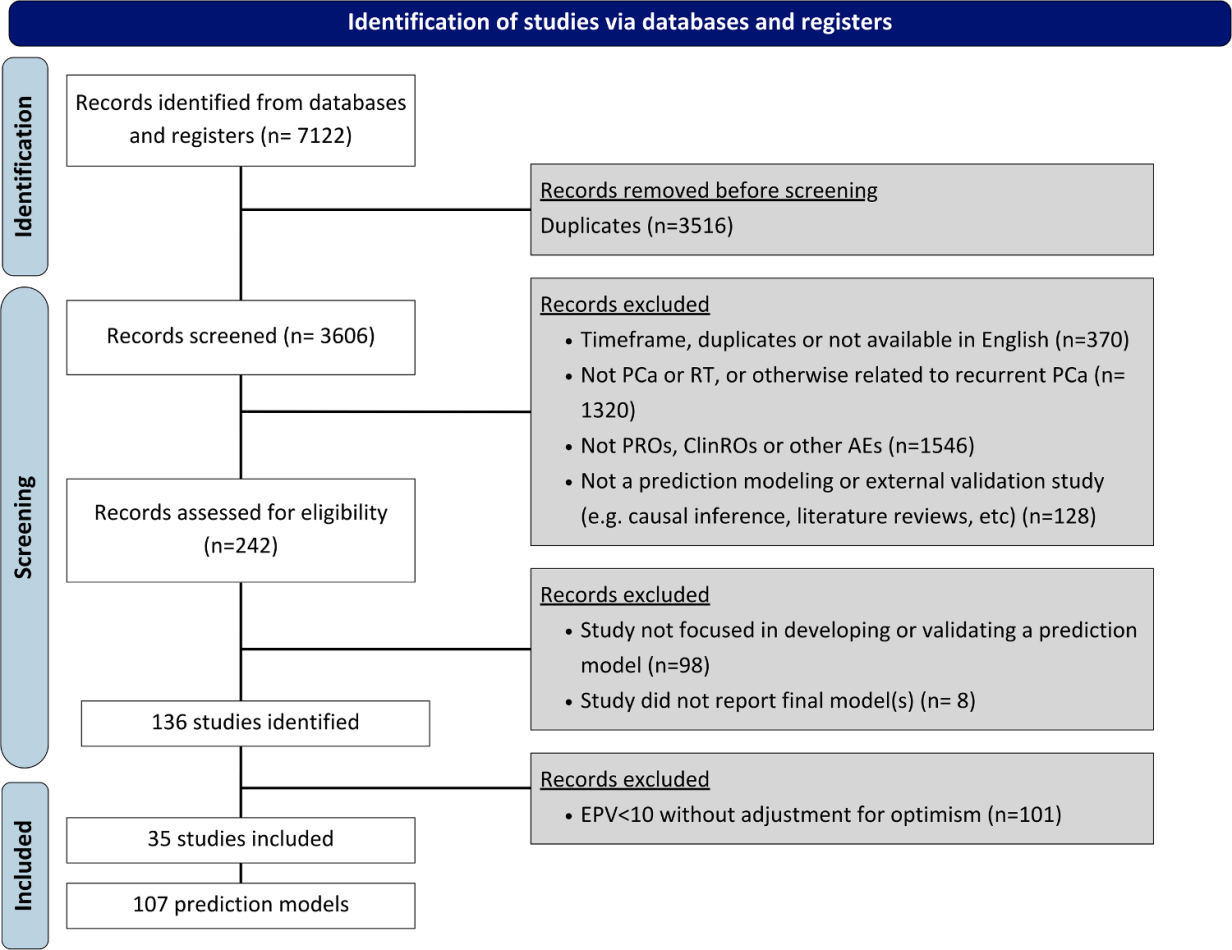
Flowchart of the literature search (PRISMA) [71]. Note: RT (Radiotherapy); PCa (Prostate Cancer); PROS (Patient-Reported Outcomes); ClinROs (Clinician-Reported Outcomes); AES (Adverse events); SVMs (Support vector machines); ANN (Artificial neural networks); EPV (Event Per Variable).

After screening the 3606 studies retrieved from the search strategy, we identified 136 CPM development or validation studies, including baseline predictors of AEs after PCa-RT (Fig. 1). Of these, we included 35 studies at low risk of optimism, comprising 107 models [[36], [37], [38], [39], [40], [41], [42], [43], [44], [45], [46], [47], [48], [49], [50], [51], [52], [53], [54], [55], [56], [57], [58], [59], [60], [61], [62], [63], [64], [65], [66], [67], [68], [69], [70]]. Of the studies included, only three were external validation studies [41,58,70].

### Characteristics of the included models

Most models (*n* = 69) predict radiation-induced AEs in the genitourinary (GU) tracts, while others predict AEs in the gastrointestinal (GI) tract (*n* = 27), erectile-related structures, which affect sexual function (*n* = 7), and bone marrow, leading to hematologic toxicity (*n* = 4; Fig. 1, Supplementary A). No models predicting health-related quality of life (HRQoL) were identified. Moreover, most of the models focused on predicting late toxicities (*n* = 66), comprising AEs that occurred between three months post-RT and up to five years after treatment completion. Only two models from a single study focused on predicting the late persistence of AEs, defined as moderate-to-severe toxicity on average over three years or as failure to fully recover after deterioration in functional outcome [43].

Different instruments were used to measure AEs. For GU outcomes, most models predicted PROs (*n* = 56), using mostly the Late Effects Normal Tissue Task Force (LENT)-Subjective, Objective, Management, Analytic (SOMA) -LENT/SOMA- scale or the International Prostate Symptom Score (IPSS) to record complications. Other PROs were less commonly used, such as the Expanded Prostate Cancer Index Composite (EPIC-26) and the Urinary Incontinence Short Form (ICIQ-UI SF). For models predicting ClinROs (*n* = 11), the Toxicity Criteria of the Radiation Therapy Oncology Group (RTOG) and the European Organization for Research and Treatment of Cancer (EORTC) - RTOG/EORTC- criteria or the Common Terminology Criteria for Adverse Events (CTCAE) were used. Finally, two models predicted the need for medical interventions due to GU toxicity, namely, urinary retention requiring catheterization and hospital admission due to GU toxicity. For GI outcomes, most models predicted ClinROs (*n* = 19), using either the RTOG/EORTC or the CTCAE criteria. For the GI models predicting PROs (*n* = 8), the LENT/SOMA scale or the Inflammatory Bowel Disease Questionnaire (IBDQ) were used. Sexual function was defined as being able to have a functional erection, and it captured the patient's experience using the EPIC-26 or the EPIC for clinical practice (EPIC-CP) questionnaires, or through a single-item measure to score erectile function as 0 if unable to attain an erection, as 1 if able to attain an erection that is either insufficient or cannot be sustained to allow for vaginal penetration, as 2 if the erection is sufficient for vaginal penetration but suboptimal or as 3 if the erectile function is considered by the patient to be normal [49]. Finally, hematologic toxicity was graded using CTCAE.

Models were either developed using subjects from prospective cohorts that aimed to study radiation-induced toxicity patterns in patients undergoing PCa-RT (*n* = 49) or otherwise from previous randomized-controlled trials (RCTs) comparing different RT regimens or types (*n* = 47). The development cohort was derived from retrospective cohort studies in 11 models. Furthermore, most studies used observational data sources from multicenter cohorts (*n* = 92).

Most models (*n* = 87) were developed on subjects that underwent EBRT, six on subjects that underwent adjuvant post-operative radiotherapy (PORT) [38,55,64], three on subjects that underwent BT [36,54,59], and the remaining 11 models were developed on varied population samples that underwent different RT types, sometimes in combination [[47], [48], [49],^63^].

### Methodological approaches

Most models predicted AEs using multivariate logistic regression (LR) (*n* = 100). Of these, six models simultaneously used texture analysis to identify 3D dosimetric predictors [60]. Random forests (*n* = 2) and support vector machines (*n* = 2) were used to develop four models [47]. Additionally, four models were developed using a Lyman-Kutcher-Burman approach (LKB) [52,65,66] and one using a Cox proportional hazards regression (CPH) [44].

In total, 11 models developed by four different studies fulfilled the criterion of EPV ≥ 10, and eight of those performed internal validation using bootstrapping [42,44,66,68]. Other prediction model developers used strategies to prevent overfitting: corrected their performance measures for optimism at internal validation with bootstrapping (*n* = 82), cross-validation (*n* = 6), or 10-fold cross-validation (*n* = 3); penalized their predictors’ strength using Least Absolute Shrinkage and Selection Operator (LASSO) (*n* = 12) [45,50,52,53,61,62,65], or a heuristic formula to derive a shrinkage factor (*n* = 1) [59]. One model used adaptive elastic net regularization to avoid optimism, while it also fulfilled the criterion of EPV ≥ 10 [44]. In total, 68 models used univariate analysis for variable selection with different significance values for selection (*p*-values = 0.05–0.2) either as a single approach (*n* = 3) or in combination with other data-driven approaches (*n* = 27). Stepwise selection (*n* = 76), LASSO (*n* = 12) [45,50,52,53,61,62,65], recursive feature selection (*n* = 22) [[47], [48], [49]] and bootstrapping (*n* = 2) [40,51] were other methods used for variable selection. Six models applied a full model approach [54,59]. Of the models included, 22 were externally validated [36,41,47,48,58,70].

### Risk of bias

**Fig. 2:**
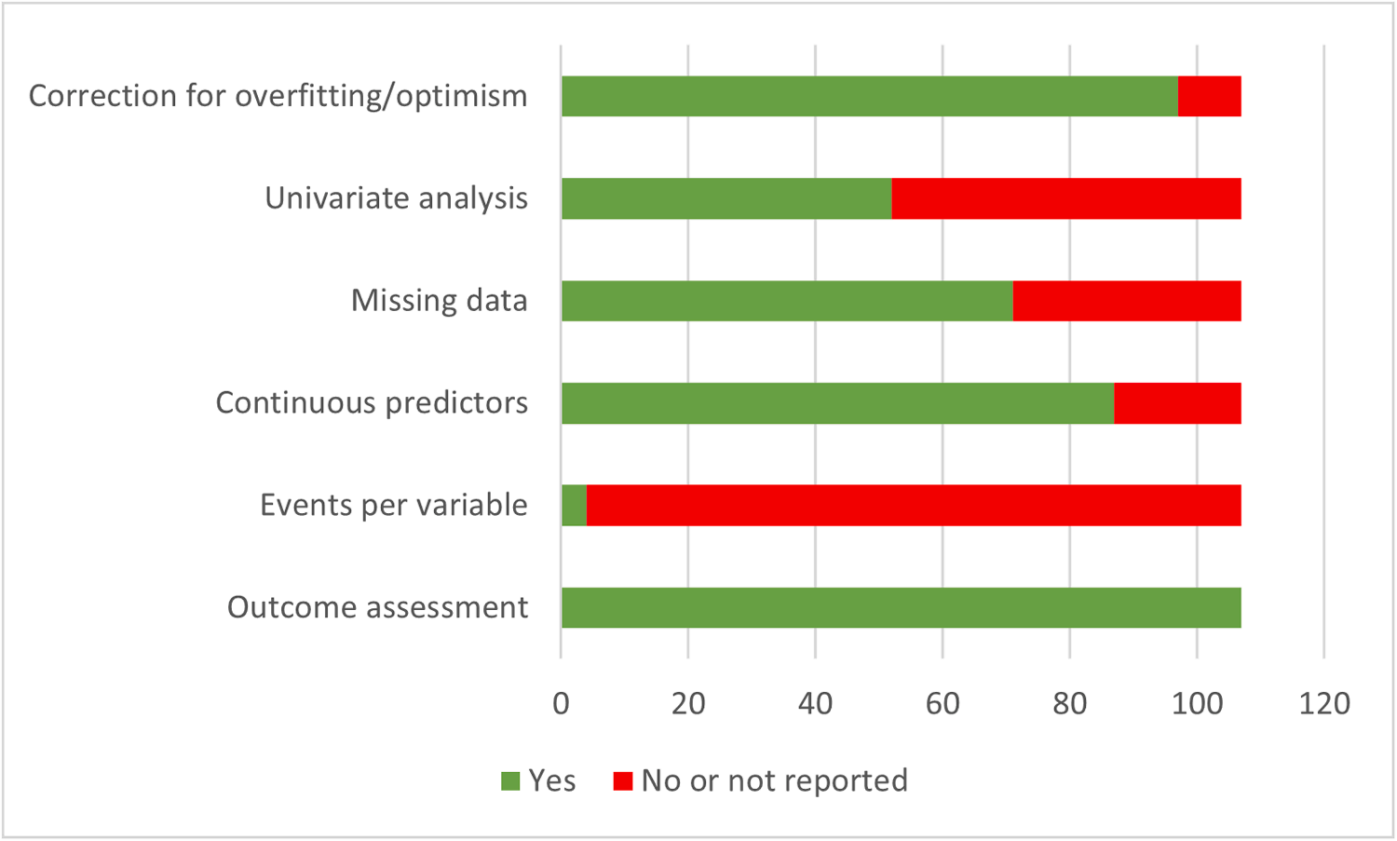
Risk of bias assessment with the short-form, per item. Note: The first item of the short form (“Outcome assessment”) belongs to domain 3 (“Outcome”) of the PROBAST tool, and all others to domain 4 (“Analysis”).

Of the 107 models, only two were classified as low-risk of bias, meeting all the criteria of the SF-PROBAST: one model predicting overall GU toxicity and one model predicting dysuria [44,69]. The remaining models were classified as high-risk of bias by the SF-PROBAST tool, mostly due to the low number of events per variable, despite having provided optimism-corrected performance or effect estimates (Fig. 2). Other common unmet criteria were using univariable analysis to select predictors and not describing the way missing data were handled. In total, 73 models met at least four out of six criteria of the SF-PROBAST.

### Performance of included prediction models

#### At development

The discriminative performance of most models was provided as the AUC or c-statistic, but 10 models reported other performance measures instead, such as R^2^ [38,43,55]. Nine models did not report any performance measures.

**Fig. 3:**
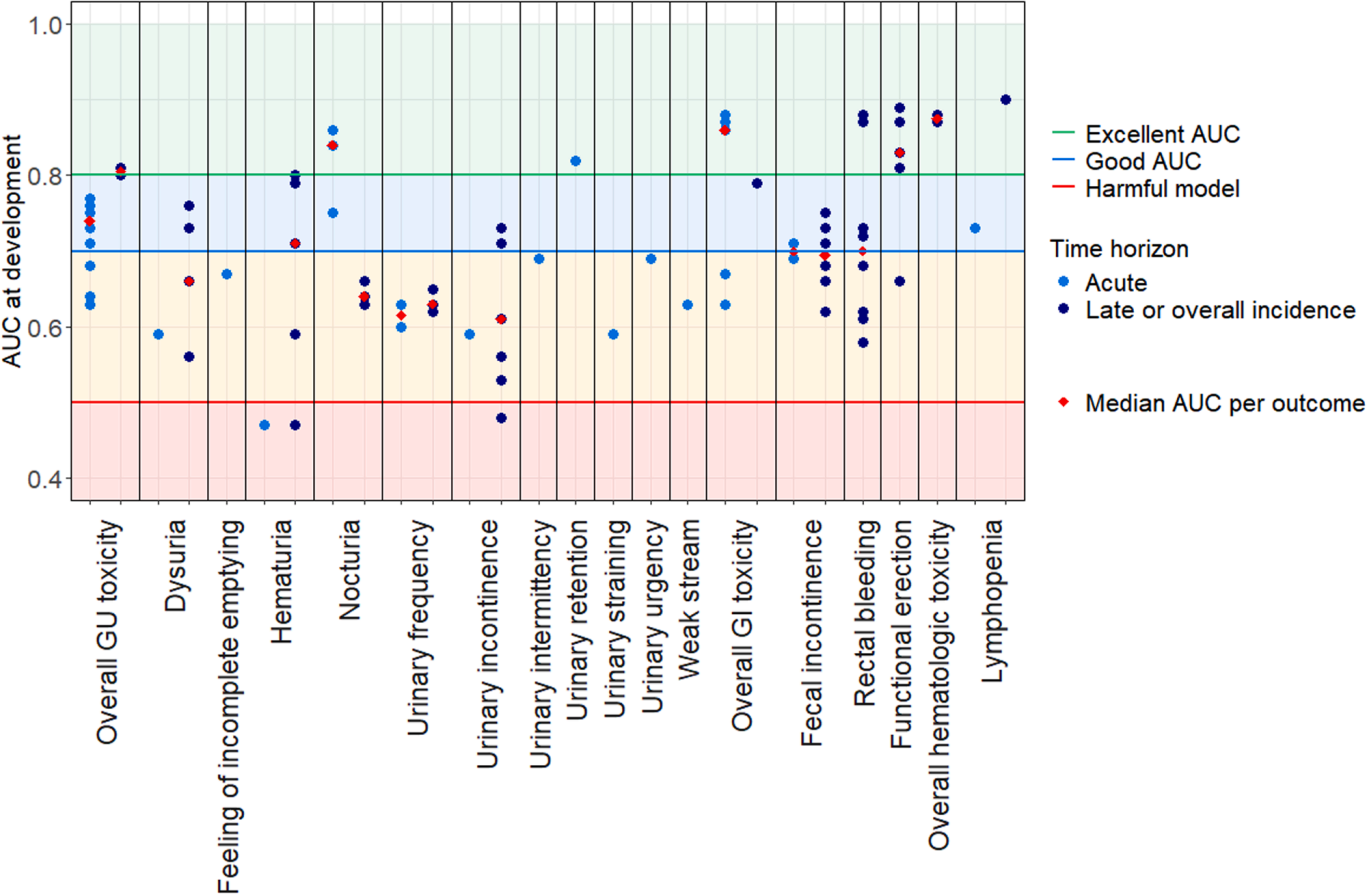
Discriminative ability of models at development per outcome. Note^1^: The points in red display the median discriminative ability of models per outcome. Note^2^: Discrimination refers to the ability of a model to distinguish between individuals at higher versus lower risk of an event. A discrimination value (e.g., C‑statistic or AUC) of 0.50 indicates no discriminative ability—equivalent to random chance—whereas a value of 1.0 represents perfect discrimination. A discriminative ability below 0.50 indicates that models predict higher risks of complications for patients that are in fact at a lower risk (harmful models).

A total of 19 CPMs developed by 12 different studies report excellent discriminative ability (AUC ≥ 0.80), such as for predicting rectal bleeding [50], overall GI toxicity [53], hematuria[45], nocturia [52], overall GU toxicity [44,51], urinary retention [59], lymphopenia [57,63], and functional erection [36,48,54] (Fig. 3). Of these, the model predicting urinary retention and the models predicting functional erection at different time points were developed on patients undergoing BT (AUCs equal to 0.82, 0.87, and 0.89, respectively) [36,54,59]. For patients undergoing PORT, one model predicting lymphopenia also reports excellent discriminative ability (AUC = 0.90) [63]. Of the aforementioned 19 models, only one assessed calibration, reporting an excellent calibration slope (0.99) [61]. Of the 107 models, 25 developed by nine studies report good discriminative ability (AUC 0.70–0.80) [39,40,42,46,56,60,62,64,69], and of those, three report good to excellent calibration slopes (1.00–1.10), while two reveal overfitting (slope < 0.80). Of the 25 models, one model predicts fecal incontinence and another predicts lymphopenia in patients undergoing PORT (AUC 0.71 and 0.73, respectively) [63,64]. Additionally, 30 of the 107 models developed by nine studies show moderate performance (AUC 0.60–0.70) [37,42,49,56,60,64,67,69,72], and of these, seven report excellent calibration slopes (around 1), while in eight models, significant overfitting is observed (slope < 0.80). A wide range of reported discriminative abilities (ΔAUC > 0.20) was observed for dysuria, hematuria, urinary incontinence, overall GI toxicity, rectal bleeding, and functional erection. A total of nine models predicting dysuria, hematuria, urinary incontinence, straining or frequency, and rectal bleeding show poor discriminative ability (AUC 0.50–0.60), and three models for hematuria and urinary incontinence show a discriminative ability below 0.50, meaning that these models predicted higher risks of complications for patients that were in fact at a lower risk. A total of 40 models report calibration slopes derived from internal validation with bootstrapping (Supplementary B), ranging between 0.43 and 1.11 [37,38,43,46,55,56,61,64,69]. Of these, 19 models developed by eight studies report calibration slopes below 0.80, revealing considerable overestimation of predictors’ strength at development (overfitting). Despite this, regression coefficient penalization was not employed in any of these models.

#### At external validation

**Fig. 4:**
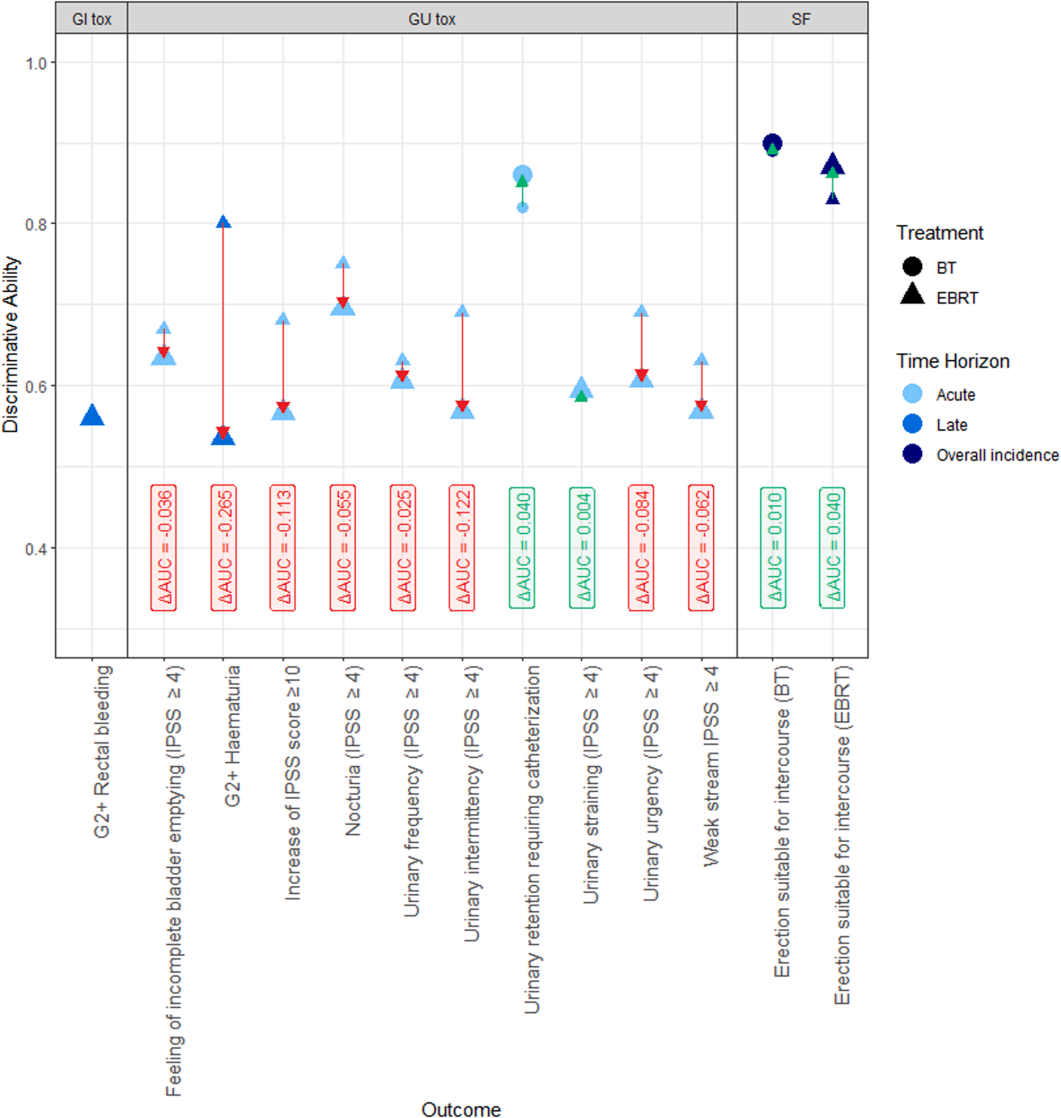
Discriminative ability at development and external validation per model. Note^1^: The arrow points at the AUC at validation. If red, the model performed worse in the external dataset than in the development dataset; if green, it performed better. Note^2^: For moderate to severe rectal bleeding (grade 2 and 3), discriminative ability at development could not be retrieved and thus, the difference in discrimination at validation could not be assessed. Note^3^: Discrimination refers to the ability of a model to distinguish between individuals at higher versus lower risk of an event. A discriminative ability below 0.50 indicates that models predict higher risks of complications for patients that are in fact at a lower risk (harmful models).

Out of the 22 externally validated models, 12 were validated in three separate studies [41,58,70], while the remaining ten models were externally validated in the development study. For the models predicting moderate to severe hematuria (grades 2 and 3) [45,70], an increase of at least 10 points in IPSS score [56,70], and an increase of at least 4 points in IPSS score for the urinary intermittency domain [42,70], the drop in discriminative ability at external validation was substantial (ΔAUC > −0.10) (Fig. 4). In contrast, for the model predicting urinary retention requiring catheterization [58,59] and the models predicting an erection suitable for intercourse [36], the discriminative ability was higher at validation (ΔAUC = 0.04, 0.04, and 0.01, respectively).

**Fig. 5:**
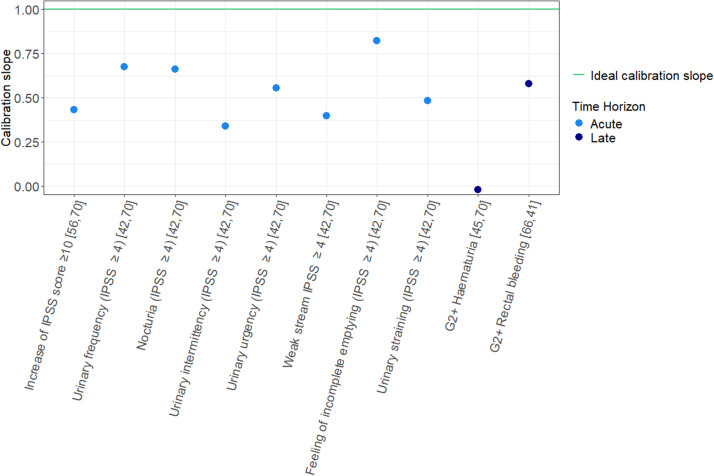
Calibration slope at external validation per model. Note: the calibration slope assesses whether the strength of the predictors—reflected in the model's coefficients—is accurately estimated in the validation cohort. A calibration slope of 1 indicates perfect calibration (green dashed line); values <1 suggest that the predictor effects are overestimated, while values greater than 1 indicate underestimation.

Calibration was assessed in 10 models across two independent validation studies [41,70], consistently revealing substantial overestimation of predictor effects (calibration slopes −0.02–0.83; Fig. 5).

## Best performing models with an acceptable methodological quality

Out of 107 models, 32 developed by 13 different studies were identified with a good discriminative ability at development (AUC ≥ 0.70) and met at least four out of six criteria from the SF-PROBAST (Table 1). Among these, only one model was classified as having a low risk of bias (ROB), meeting all SF-PROBAST criteria [44]. The remaining 31 models were classified as high ROB, primarily because of an insufficient number of events per variable (EPV < 10). Furthermore, 28 of these models failed an additional SF-PROBAST criterion, either due to predictor selection based on univariate analyses (*n* = 21) or dichotomization of continuous predictors (*n* = 7) (Table 3; Supplementary Material A). Of the 32 models, five report calibration slopes from internal validation, which ranged from 0.59 to 1.11. A total of 10 out of these 32 models were externally validated, demonstrating good discriminative ability in independent cohorts (AUC ≥ 0.70). However, one of the models was assessed in terms of calibration, and it revealed that the strength of predictors was overestimated (calibration slope = 0.66). Of the 32 models, 13 models include only pre-treatment predictors, while the remaining 19 models are NTCP models, including both dosimetric and pre-treatment predictors.

**Table 1:** Best performing models (AUC ≥ 0.70) with an acceptable methodological quality (at least four out of six SF-PROBAST criteria met).

Studies Validation Development	Externally validated before? (Setting)	Outcome			*Treatment*		Predictors	*Date of inclusion* *Validation* *Development*	*Nr patients (Nr Events)* *Validation* *Development*	*Model Characteristics*	
		*Time horizon*	*Description*	*Criteria*	*RT type* *Validation* *Development*	*Prescribed dose* *Validation* *Development*				*Model*	*Discrimination* *Validation* *Development*
David 2023 [44]	*No*	Overall incidence	G2+ urinary toxicity	hospital admission with treatment-related GU	Unspecified	Unspecified	Diabetes, Smoking status, BOO without TURP and BOO with TURP	1998–2019	3243 (644)	CPH	Censor-adjusted c-statistic = 0.80
Huang 2019 [50]	No	Late	G2+ rectal bleeding	CTCAE	IMRT	72	Platelet count, D’Amico risk classification, Rectum V65*^1^	2008–2011	68 (15)	LR	AUC = 0.87
Rossi 2018 [60]	No	Late	Laser treatment or transfusion required after rectalbleeding	RTOG-EORTC	3D-CRT, Hypo 3D-CRT	64.6 (HYPO) or 78 (CONV)	Gleason score, LRHGE *^1^; GLN *^1^; NGTDM*^1^	2007–2010	395 (NR)	Texture analysis and LR	AUC = 0.73
Rossi 2018 [60]	No	Late	Laser treatment or transfusion required after rectal bleeding	RTOG-EORTC	3D-CRT, Hypo 3D-CRT	64.6 (HYPO) or 78 (CONV)	Gleason score, Rectum V55 *^1^, LRHGE *^1,^ GLN*^1^; NGTDM*^1^	2007–2010	395 (NR)	Texture analysis and LR	AUC = 0.72
Rossi 2018 [60]	No	Late	Use of diapers due to fecal incontinence	RTOG-EORTC	3D-CRT, Hypo 3D-CRT	64.6 (HYPO) or 78 (CONV)	Age, LRHGE *^1^, RLV*^1^, NGTDM*^1^	2007–2010	395 (NR)	Texture analysis and LR	AUC = 0.75
Rossi 2018 [60]	No	Late	Use of diapers due to fecal incontinence	RTOG-EORTC	3D-CRT, Hypo 3D-CRT	64.6 (HYPO) or 78 (CONV)	Age, Rectum Dmin*^1^, LRHGE *^1^, RLV*^1^, NGTDM*^1^	2007–2010	395 (NR)	Texture analysis and LR	AUC = 0.75
Rossi 2018 [60]	No	Late	Use of diapers due to urinary incontinence	RTOG-EORTC	3D-CRT, Hypo 3D-CRT	64.6 (HYPO) or 78 (CONV)	Baseline symptoms, Age, prostate volume, TURP, GI comorbidities	2007–2010	395 (NR)	Texture analysis and LR	AUC = 0.71
Rossi 2018 [60]	No	Late	Use of diapers due to urinary incontinence	RTOG-EORTC	3D-CRT, Hypo 3D-CRT	64.6 (HYPO) or 78 (CONV)	Baseline symptoms, Age, TURP, GI comorbidities, Bladder V35 *^1^	2007–2010	395 (NR)	Texture analysis and LR	AUC = 0.71
Rossi 2018 [60]	No	Late	Use of diapers due to urinary incontinence	RTOG-EORTC	3D-CRT, Hypo 3D-CRT	64.6 (HYPO) or 78 (CONV)	Baseline symptoms, Age, TURP, GI comorbidities, Bladder V35 *1, LRHGE*1, NGTDM*1	2007–2010	395 (NR)	Texture analysis and LR	AUC = 0.72
Sini 2017 [64]	No	Acute	Loose stools: ΔIBDQ5 < −2 (maximum variation)	IBDQ	PORT WPRT	66.6–78, pelvic nodes (50.4–54) or 70–80, pelvic nodes (50.4–54.4)	Age, DVH shape *^1^	2012–NR	216 (43)	LR	AUC = 0.71
Carillo 2014 [39]	No	Acute*	IPSS ≥ 15	IPSS	IMRT, Hypo-IMRT with or without WPRT	74–80 (CONV) or 65–75.2 (HYPO)	Baseline score, Antihypertensive drugs, Clinical stage, Bladder aS8.5w and aS12.5w*^1^	2010–2014	247 (77)	LR	AUC = 0.76
Carillo 2014 [39]	No	Acute*	IPSS ≥ 15	IPSS	Hypo-IMRT with or without WPRT	65–75.2 (HYPO)	Baseline score, Antihypertensive drugs, Clinical stage, Bladder aV8.5w*^1^ and aV12.5w*^1^	2010–2014	247 (77)	LR	AUC = 0.75
Carillo 2014 [39]	No	Acute*	IPSS ≥ 15	IPSS	IMRT, Hypo-IMRT with or without WPRT	74–80 (CONV) or 65–75.2 (HYPO)	Baseline score, Antihypertensive drugs, Clinical stage, Bladder aV80*^1^	2010–2014	247 (77)	LR	AUC = 0.76
Carillo 2014[39]	No	Acute*	IPSS ≥ 15	IPSS	Hypo-IMRT with or without WPRT	65–75.2 (HYPO)	Baseline score, Antihypertensive drugs, Clinical stage, Bladder aS80*^1^	2010–2014	247 (77)	LR	AUC = 0.76
Carillo 2014 [39]	No	Acute*	IPSS ≥ 15	IPSS	IMRT, Hypo-IMRT with or without WPRT	74–80 (CONV) or 65–75.2 (HYPO)	Baseline score, Antihypertensive drugs, Clinical stage, Bladder aS8.5w*^1^ and aS12.5w*^1^	2010–2014	131 (61)	LR	AUC = 0.73
Carillo 2014 [39]	No	Acute*	IPSS ≥ 15	IPSS	Hypo-IMRT with or without WPRT	65–75.2 (HYPO)	Baseline score, Antihypertensive drugs, Clinical stage, Bladder aV8.5w*^1^ and aV12.5w*^1^	2010–2014	131 (61)	LR	AUC = 0.73
Carillo 2014[39]	No	Acute*	IPSS ≥ 15	IPSS	IMRT, Hypo-IMRT with or without WPRT	74–80 (CONV) or 65–75.2 (HYPO)	Baseline score, Antihypertensive drugs, Clinical stage, Bladder aV60*^1^ and aV80*^1^	2010–2014	131 (61)	LR	AUC = 0.75
Carillo 2014 [39]	No	Acute*	IPSS ≥ 15	IPSS	Hypo-IMRT with or without WPRT	65–75.2 (HYPO)	Baseline score, Antihypertensive drugs, Clinical stage, Bladder aS60*^1^ and aS80*^1^	2010–2014	131 (61)	LR	AUC = 0.77
Palorini 2016 [56]	No	Acute	IPSS ≥ 15	IPSS	Hypo-IMRT with or without WPRT	65–75.2 (HYPO)	ADT, Antihypercholesterolemia Drugs × Absolute Weekly DSH at 12Gy cm² *^1^, cardiovascular drugs × Absolute Weekly DSH at 12Gy cm² *^1^	2003–2007	380 (28)	LR	AUC = 0.71
Yahya 2015 [69]	No	Late*	G1+ Dysuria	LENT-SOMA	3D-RT	66, 70, 74	Baseline score, NSAIDS, NIDDM, Age, Cerebrovascular disorders, ACE inhibitor, Statins, Respiratory disorders, Anticoagulants	2003–2007	754 (28)	LR	AUC = 0.73
Yahya 2015 [69]	No	Late*	G1+ Hematuria	LENT-SOMA	3D-RT	66, 70, 74	Baseline score, NIDDM, PC1	2003–2007	754 (14)	LR	AUC = 0.79
Same study Hasannejadasl 2023 [48]	Different hospitals from the same country	Late*	Erectile dysfunction	EPIC-26	EBRT or BT	Varied (national cohort)	PCa treatment, quality of erections, frequency of erections, ISUP, clinical stage, ADT, cardiovascular diseases, diabetes, Lack of energy, alcohol use	2015–2016 2015–2016	Development + Validation: 848 (390)	LR	AUC = 0.84 NR
Same study Hasannejadasl 2023 [48]	Different hospitals from the same country	Late*	Erectile dysfunction	EPIC-26	EBRT or BT	Varied (national cohort)	Quality of erections, frequency of erections, treatment, ISUP, clinical stage, CCI, ADT, Diabetes, Abdominal/pelvic/rectal pain	2015–2016 2015–2016	Development + Validation: 670 (308)	LR	AUC = 0.81 NR
Same study Hasannejadasl 2023 [47]	Different hospitals from the same country	Late*	Urinary incontinence	EPIC-26	EBRT or BT	Varied (national cohort)	Bloody stool, Leaking urine, IFBM, Urinary function, Urine loss, Diabetes, cardiovascular diseases, ADT, PCa treatment	2015–2016 2015–2016	614 (NR) 234 (NR)	LR	AUC = 0.79 NR
Same study Hasannejadasl 2023 [47]	Different hospitals from the same country	Late*	Urinary incontinence	EPIC-26	EBRT or BT	Varied (national cohort)	Type PCa treatment, PSA, Age, leaking urine, sexual function, urinary control, cT, urinary function, urine loss, CVD	2015–2016 2015–2016	614 (NR) 234 (NR)	RF	AUC = 0.74 NR
Same study Hasannejadasl 2023 [47]	Different hospitals from the same country	Late*	Urinary incontinence	EPIC-26	EBRT or BT	Varied (national cohort)	PSA, Age, type PCa treatment, CT, leaking urine, need to urinate frequently, urinary function, urinary control, sCN	2015–2016 2015–2016	479 (NR) 191 (NR)	RF	AUC = 0.71 NR
Same study Hasannejadasl 2023 [47]	Different hospitals from the same country	Late*	Urinary incontinence	EPIC-26	EBRT or BT	Varied (national cohort)	Type PCa treatment, leaking urine, urinary control, urinary function, urine loss, urinary frequency, Age, sCT, sexual functioning	2015–2016 2015–2016	614 (NR) 234 (NR)	SVM	AUC = 0.76 NR
Same study Hasannejadasl 2023 [47]	Different hospitals from the same country	Late*	Urinary incontinence	EPIC-26	EBRT or BT	Varied (national cohort)	Type PCa treatment, sCT, leaking urine, urinary control, urinary function, urinary frequency, urine loss, gleason group, sCN	2015–2016 2015–2016	479 (NR) 191 (NR)	SVM	AUC = 0.72 *NR*
Yahya 2016 Cozzarini 2015 [42,70]	Different countries	Acute*	Nocturia (IPSS ≥ 4)	IPSS	3D-CRT SD-IMRT, Hypo-IMRT, with or without IGRT	66–74 74–80 (CONV) or 65–75.2 (HYPO)	Baseline score, Bladder aS11.5w*	2003–2007 2010–2014	643 (260) 229 (42)	LR	AUC = 0.70 AUC = 0.75
Roeloffzen 2012 Roeloffzen 2011 [58,59]	Different cohorts from the same country	Acute	Urinary retention requiring catheteriza- tion	Medical records	BT I-125 BT I-125	145 145	PV, IPSS, ADT, extent of prostate protrusion	2003–2008 2005–2008	715 (67) 714 (57)	LR	AUC= 0.86 AUC= 0.82
Same study Alemozaffar 2011 [36]	Different cohorts from the same country	Late*	Erection suitable for intercourse	EPIC-26	NR NR	NR NR	Baseline score, PSA, ADT	NR 2003–2006	247 (NR) 229 (84)	LR	AUC 0.87 AUC 0.83
Same study Alemozaffar 2011 [36]	Different cohorts from the same country	Late*	Erection suitable for intercourse	EPIC-26	NR NR	NR NR	Baseline score, Age, Race, BMI	NR 2003–2006	358 (NR) 247 (107)	LR	AUC 0.90 AUC 0.89

Note*: refers to outcomes measured at a single time point (prevalence), while others reflected the incidence of AEs.

Note*1: Not a baseline predictor. Refers to either a dosimetric, texture analysis or treatment-related predictors.

Abbreviations: ACE (angiotensin-converting enzyme); ADT (androgen deprivation therapy); AUC (Area under the curve); BMI (Body Mass Index); BOO (bladder outlet obstruction); BT (Brachytherapy); CCI (Charlson comorbidity index); CPH (cox proportional hazards); CTCAE (Common Terminology Criteria for Adverse Events); conv (conventional); GLN (Grey Level Nonuniformity); GI (gastrointestinal adverse events); GU (genitourinary adverse events); Hypo (hypofractionated); ICIQ-SF (International Consultation on Incontinence Questionnaire); IMRT (intensity modulated radiotherapy); IPSS (International Prostate Symptom Score); ISUP ((International Society of Urological Pathology); (LENT-SOMA (Late Effects Normal Tissue–Subjective, Objective, Management, Analytic); LRHGE (Long Run High Grey Level Emphasis); LR (multivariate logistic regression); NGTDM (Contrast in Neighborhood Gray Tone Difference Matrix); NIDDM (Non-insulin-dependent diabetes mellitus), NR (Not reported); NSAIDS (Non-steroidal anti-Inflammatory drugs); OLR (ordinal multivariate logistic regression); PCa (Prostate cancer); PV (Prostate volume); RLV (Run Length Variance); RF (Random forests); RT (radiotherapy); SVM (Support vector machines); TURP (transurethral resection of the prostate); WPRT (whole-pelvis radiotherapy); 3D-CRT (conformal external beam radiotherapy).

Dosimetric predictors: aSx = the absolute surface receiving more than X Gy, aSxw = the absolute surface receiving more than X Gy per week, aVx = the absolute volume receiving more than X Gy, aVxw = the absolute volume receiving more than X Gy per week, Vx = the relative volume exposed to a dose of ≥X Gy, PC: Principal components of bladder dose-surface histogram.

### Baseline predictors importance

Of the included 107 models, 38 are pre-treatment models – 20 were developed with baseline predictors only, and 18 only included baseline predictors in their final models after dosimetric predictors were excluded during variable selection. The remaining 69 out of the 107 models are NTCP models, including both baseline and dosimetric predictors.

Baseline score or symptoms were consistently included as predictors for most outcomes, while comorbidities and medication use related to the affected tissues were also often included as predictors of the tissue-specific RT toxicity (Tables 1 and 2, Supplementary A). For example, the use of anticoagulants often predicted rectal bleeding, while the use of cardiovascular drugs or antihypertensives and prostate volume predicted urinary symptoms. Other common predictors for GI outcomes included age, previous surgery (TURP/abdominal), antihypercholesterolemic drugs, platelet count, and hemorrhoids. For GU outcomes, a history of transurethral resection of the prostate (TURP), RT type (PORT, BT, or EBRT), and clinical staging were often selected. For sexual outcomes, no other important predictors were identified besides baseline symptoms, while for hematologic toxicity, the absolute lymphocyte count (ALC) at baseline and smoking status were often selected. Tumor characteristics such as PSA, Gleason score, and risk group were often tested as candidate predictors but rarely selected in final models.

### Other findings related to predictor importance

Aside from the frequently selected predictor – treatment type (EBRT or PORT)– on GU outcomes, other treatment-related characteristics, such as RT prescribed dose or type, the irradiation of pelvic nodes or seminal vesicles, and androgen deprivation therapy (ADT) were often tested as candidate predictors but rarely selected in final models.

## Discussion

To our knowledge, this is the first systematic review focused on assessing the methodological soundness of models including baseline predictors for predicting AEs among patients undergoing PCa-RT, and it captured 20 years of research in the field. Models validated in terms of calibration in external datasets revealed miscalibration. Taken together with the identified risk of bias in most models, these findings indicate that none of the currently available models are ready to be implemented in clinical practice. However, we highlight 32 models for their good performance at development or in independent cohorts that did not violate more than two out of six criteria of the SF-PROBAST, to be further externally validated and, if necessary, recalibrated. Nevertheless, other models may also warrant validation, as even models with modest discriminative ability may provide clinical value, provided they are well calibrated and demonstrably improve clinical decision-making [73,74]. To facilitate future validation efforts, we provide a comprehensive overview of all included studies, including their methodologic approaches, performance, model specification, and ROB assessment (Supplementary B and C). However, it is important to note that neither of the included CPMs can be used to decide whether patients are suitable for RT. There were no study populations randomized to different first-line treatments for prostate cancer patients, and therefore, such a conclusion cannot be made from these models. These models may, however, share insights into individual risks of complications, which may support how RT can be tailored to a patient at a higher risk of GU, GI, or hematologic toxicities, and inform patients on what to expect and how to prepare during and after their RT journey.

A review published in 2017 by O’Callaghan et al. focused on the accuracy and validity of models predicting PROs after PCa-RT [75]. Considering the growing interest in moving towards precision medicine in this field, many research initiatives have focused on developing these models over the last years, and we identified 47 models since the final inclusion date for this previous review. Interestingly, we can note that since this publication by O’Callaghan et al., there is still a lack of CPMs for patients undergoing BT and PORT, or models predicting HRQoL. This may be due to the difficulty in predicting HRQoL, which is a complex measure that is highly influenced by individual perceived health and coping mechanisms [76,77]. On the other hand, O’Callaghan et al. used a different tool to critically appraise the included prediction modeling studies [78]. While this assessment focused mainly on predictors and outcome-related sources of bias, it overlooked analysis-related sources of bias, which were the main source of bias recognized in previous systematic reviews of prediction modeling studies [25,79,80].

### The need for external validation

The generalizability of a prediction model refers to the ability to make predictions for different populations than the one used for model development, in terms of time (predictions in future patients) and space (predictions in different regions) [81]. Assessing calibration at external validation is imperative to ascertain whether models can make accurate predictions, but it was rarely considered by the included validation studies. Even though most models seemed to perform adequately in terms of discriminative ability in external datasets, the observed calibration slopes of the few models assessing calibration revealed that these were not generalizable, underlying the importance of this assessment. Previous studies in various fields have highlighted the difficulty in identifying promising models for implementation in clinical practice, considering the vast number of existing models and the low number of external validations to confirm the validity of their predictions [[82], [83], [84]]. There is a pressing need for additional external validation studies, while new model development should only be pursued if no suitable CPMs are available [83,85,86]. Notably, datasets potentially insufficient to develop a comprehensive model at low risk of bias may still be sufficient to share insights on how a model performs in independent datasets, or for model updating purposes [[87], [88], [89]].

### Methodological recommendations for future model development

This review has shown that most models in the PCa-RT field were developed on limited sample sizes. The few calibration assessments observed during both internal and external validation suggest that these models are prone to overfitting. Consequently, their predictions may be poorly calibrated, limiting their reliability and hindering their implementation in clinical practice [31,72]. For future model development, combining similar outcomes into composite outcomes can increase effective sample sizes and help prevent overfitting, which may improve model generalizability. If uncommon events are relevant to predict, larger datasets should be used. Furthermore, a more rigorous minimum sample size calculation is advised, as the commonly used criterion of EPV > 10 has been shown to underestimate the true sample size requirements [90]. Several models predicting the same outcome were identified, often with wide variability in discriminative performance. Combining multiple datasets can simultaneously increase sample sizes and sample heterogeneity—an advantage for enhancing the generalizability of predictive models [91). To do so, it is important to harmonize the use of criteria to record toxicity and complications across cohorts [80,91,92]. Furthermore, most of the tested predictors seem to be irrelevant at predicting complications, except for baseline symptoms/toxicity and comorbidities or medication-use that influence radio-sensitive organs. We recommend a more parsimonious inclusion of clinical predictors by pre-selecting candidate variables based on clinical knowledge, as well as using data-driven variable selection and predictor strength penalization methods (e.g., bootstrapping, LASSO, elastic net regularization) to further mitigate the risk of overfitting [72,93]. We also highlight the importance of avoiding variable selection using univariate analysis, which may lead to the elimination of predictors relevant in a multivariate context [94,95]. Finally, while several models may imply an excellent predictive ability at development, this may merely be a symptom of overfitting. Performing internal validation using bootstrapping or 10-fold cross-validation is crucial to assess and correct performance measures for optimism [85]. While most identified models fulfilled this step, the reporting of the calibration slope was often missed. For the models that report a calibration slope below 0.80, it is crucial to penalize the coefficients to correct for model optimism before applying the model in practice [72]. Nevertheless, the generalizability of prediction models for PROs, ClinROs, and complications after prostate cancer RT may be determined by differences in treatment planning techniques and fractionation schemes. These differences remain influential even when dosimetric predictors are included, given the constrained reliability of their measurement [[96], [97], [98]]. For this reason, when applying prediction models to a new setting, these should always be first externally validated and recalibrated if necessary [99]. Similar to other medical fields, the reporting in the prediction modeling studies was often inadequate [84,100]. Specifically, machine learning studies rarely report their final models or provide the necessary materials to reproduce the trained models, which hinders their evaluation and use. We recommend that the TRIPOD+AI reporting guidelines be followed to foster the external validation and implementation of CPMs [101].

### Limitations

Several CPMs were excluded due to a high risk of optimism at development. This criterion (EPV<10 without providing optimism-corrected performance measures or effect estimates) relies on a threshold that cannot fully capture the actual risk of optimism of different models. Therefore, such a criterion may have led to the inclusion of overfitted models, wrongly classified as low risk of optimism, or the exclusion of relevant models that would have perhaps been generalizable. This review also excluded longitudinal models, potentially overlooking relevant models in the PCa-RT field**.** The risk of bias assessment was based on a short form, which includes a subset of questions presented in PROBAST. Even though this form has been previously validated and shown to provide similar risk of bias assessments to the PROBAST itself [35], some sources of bias could have been missed in this review.

### Considerations for developing and identifying relevant prediction models for the prostate cancer radiotherapy care path

There is an untapped potential in the use of CPMs to support PCa- RT care paths [12,102]. Most of the included models appear clinically applicable, given their appropriate selection of outcomes and predictors, and the methods used to measure these. Regarding the study populations, it is important to note that many models were developed or validated using data from RCTs, which often include highly selected patient populations. As a result, these development cohorts may not fully reflect real-world clinical populations, potentially limiting model transportability and generalizability.

On the other hand, identifying which models are truly suitable for clinical implementation requires further nuanced consideration. While the relevance of CPMs for treatment planning has been recognized, more research is needed to understand which predictors are relevant to accurately estimate the risks of complications and further individualize treatment [12,103]. Identifying relevant outcomes that aid decision-making and set realistic patient expectations is crucial to identifying which models may be of value for clinical implementation. Predicting the risk of acute events is important, since there is evidence supporting that patients experiencing these events are at a higher risk of experiencing late events [104]. The prediction of late events is crucial since these can be chronic and may lead to quality-of-life deterioration in cancer survivors [105]. While the incidence of events may support tailored treatment planning, the prevalence or persistence of symptoms of late outcomes may carry more relevant information to patients; however, persistence of late outcomes was found to be uncommonly predicted [106]. Furthermore, studies comparing ClinROs and PROs have noted a mismatch between patients' and clinicians' perceptions of symptom severity, and underreporting of toxicity when using ClinROs [57]. PROs instruments must then be used to capture the real impact of RT on patients, especially when these predictions are meant to inform patients. Even after assessing the identified models in terms of validation and clinical utility, the decision on whether a model is suitable for implementation is based on who the model would inform (outcome), if predictors are readily available at the moment the model would be used (predictors), if the population and the treatment regimens are similar to the ones used for model developed or validation (study design) and whether the model would improve decision-making beyond other existing decision frameworks (decision analytics) [107,108].

## Conclusion

Ready-to-use CPMs in the field of PCa-RT are very limited, while there is clearly a clinical need for predicting individualized risks of AEs. Most of the studies, including recently published work, failed to follow previous recommendations for model development and were not adequately reported based on TRIPOD+AI guidelines. Several models predicting urinary, bowel and sexual AEs were identified as good-performing and methodologically sound but should undergo further validation. Future studies should prioritize external validation and update of existing models over developing new models. In case new models are developed, larger datasets and correction for model optimism should be applied.

## Funding sources

CCO and DvK are funded by the European Union under Horizon Europe Work Programme 101057332. Views and opinions expressed are however those of the author(s) only and do not necessarily reflect those of the European Union or the European Health and Digital Executive Agency (HaDEA). Neither the European Union nor the granting authority can be held responsible for them. The UK team are funded under the Innovate UK Horizon Europe Guarantee Programme, UKRI Reference Number: 10041120.

**Figure:**
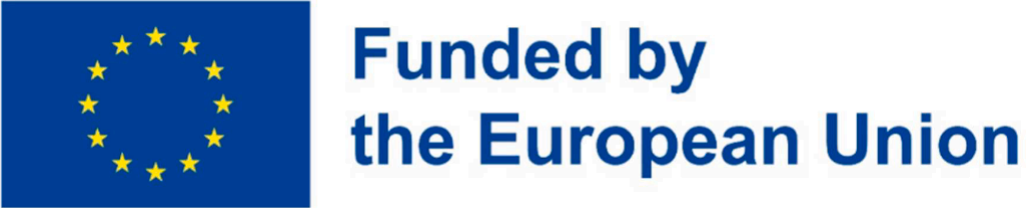

## Data statement

All data are provided in the supplementary materials A, B and C.

## CRediT authorship contribution statement

**Claudia Cruz Oliveira:** Writing – original draft, Formal analysis, Conceptualization. **Christian A M Jongen:** Writing – review & editing, Formal analysis, Conceptualization. **Ana Mikolić:** Writing – review & editing, Methodology, Conceptualization. **Luca Incrocci:** Writing – review & editing. **Hester F Lingsma:** Writing – review & editing. **Monique J Roobol:** Writing – review & editing. **Ida Korfage:** Writing – review & editing. **Wilma D Heemsbergen:** Writing – review & editing, Supervision, Methodology, Conceptualization. **David van Klaveren:** Writing – review & editing, Supervision, Methodology, Conceptualization.

## Declaration of competing interest

The authors have no conflicts of interest to declare.

## Appendix Supplementary materials

Image, application 1

Image, application 2

Image, application 3
