# Supplementary material for: Prediction models for adverse events from prostate cancer curative radiotherapy: a systematic review of methodological quality
Source: Transl Oncol. 2026 Aug 8;72:102961. doi: 10.1016/j.tranon.2026.102961 (PMC13476466; doi:10.1016/j.tranon.2026.102961)
Supplement: Supplementary file 1 [file mmc1.docx]

**Supplementary material A**

Prediction models for patient or clinician-reported outcomes and complications among patients with localized prostate cancer undergoing radiotherapy with curative intent: A systematic review

[1.	Search strategy	2](#_Toc235136087)

[2.	TRIPOD SRMA-checklist for reporting systematic reviews of prediction model studies	5](#_Toc235136088)

[3.	Figure displaying outcome distribution of the included studies	8](#_Toc235136089)

[4.	Supplementary tables	9](#_Toc235136090)

[5.	Risk of bias assessment with the short -form (including explanation)	19](#_Toc235136091)

[6. References	27](#_Toc235136092)

## 1. Search strategy

| **Database searched** | **Platform** | **Years of coverage** | **Records** | **Records after duplicates removed** |
| --- | --- | --- | --- | --- |
| Medline ALL | Ovid | 1946 - Present | 1828 | 1826 |
| Embase | Embase.com | 1971 - Present | 2361 | 696 |
| Web of Science Core Collection* | Web of Knowledge | 1975 - Present | 2420 | 773 |
| Cochrane Central Register of Controlled Trials | Wiley | 1992 - Present | 313 | 228 |
| Additional Search Engines: Google Scholar** (200 top-ranked) | | | 200 | 83 |
| **Total** | | | **7122** | **3606** |

*Science Citation Index Expanded (1975-present) ; Social Sciences Citation Index (1975-present) ; Arts & Humanities Citation Index (1975-present) ; Conference Proceedings Citation Index- Science (1990-present) ; Conference Proceedings Citation Index- Social Science & Humanities (1990-present) ; Emerging Sources Citation Index (2005-present)

**Google Scholar was searched via "Publish or Perish" to download the results in EndNote.

No other database limits were used than those specified in the search strategies

**Embase**

('prostate tumor'/exp OR (((prostat*) NEAR/3 (tumor* OR tumour* OR neoplas* OR carcino* OR cancer* OR malignan* OR adenoma* OR adenocarcinoma* OR radiotherap* OR brachytherap*))):ab,ti,kw) **AND** (radiotherapy/exp OR radiotherapy/lnk OR 'iodine 125'/de OR irradiation/de OR (radiotherap* OR brachytherap* OR radiation* OR irradiat* OR ((radio* OR irradiat* OR proton* OR arc) NEAR/3 (therap* OR treatment*)) OR EBRT OR iodi*-125* OR I125 OR I-125 OR 125I OR 125-I):ab,ti,kw) **AND** ('predictive model'/de OR nomogram/de OR 'artificial neural network'/exp OR prediction/mj/de OR 'prediction and forecasting'/mj/de OR (((predict* OR forecast* OR prognos*) NEAR/3 (model* OR score OR scores OR index OR equation* OR formul* OR tool*)) OR nomogram* OR ((neural) NEAR/3 (network*))):ab,ti,kw OR predict*:ti) NOT ([animals]/lim NOT [humans]/lim) NOT ([Conference Abstract]/lim OR [Conference Review]/lim) NOT (metastasis/mj/de OR 'second cancer'/mj/de OR radioligand/mj/de OR (metasta* OR second-canc* OR radioligand*):ti) NOT ('editorial'/de)

**Medline**

(exp Prostatic Neoplasms/ OR (((prostat*) ADJ3 (tumor* OR tumour* OR neoplas* OR carcino* OR cancer* OR malignan* OR adenoma* OR adenocarcinoma* OR radiotherap* OR brachytherap*))).ab,ti,kf.) **AND** (exp Radiotherapy/ OR radiotherapy.fs. OR Iodine-125.nm. OR (radiotherap* OR brachytherap* OR radiation* OR irradiat* OR ((radio* OR irradiat* OR proton* OR arc) ADJ3 (therap* OR treatment*)) OR EBRT OR iodi*-125* OR I125 OR I-125 OR 125I OR 125-I).ab,ti,kf.) **AND** (exp Nomograms/ OR exp Neural Networks, Computer/ OR *Forecasting/ OR *Prognosis/ OR (((predict* OR forecast* OR prognos*) ADJ3 (model* OR score OR scores OR index OR equation* OR formul* OR tool*)) OR nomogram* OR ((neural) ADJ3 (network*))).ab,ti,kf. OR predict*.ti.) NOT (exp Animals/ NOT Humans/) NOT (news OR congres* OR abstract* OR book* OR chapter* OR dissertation abstract*).pt. NOT (exp *Neoplasm Metastasis/ OR *Neoplasms, Second Primary/ OR *Radioligand Assay/ OR (metasta* OR second-canc* OR radioligand*).ti.) NOT (editorial).pt.

**Cochrane**

(((((prostat*) NEAR/3 (tumor* OR tumour* OR neoplas* OR carcino* OR cancer* OR malignan* OR adenoma* OR adenocarcinoma* OR radiotherap* OR brachytherap*))):ab,ti) **AND** ((radiotherap* OR brachytherap* OR radiation* OR irradiat* OR ((radio* OR irradiat* OR proton* OR arc) NEAR/3 (therap* OR treatment*)) OR EBRT OR iodine-125* OR iodide-125* OR I125 OR 125I):ab,ti) **AND** ( (((predict* OR forecast* OR prognos*) NEAR/3 (model* OR score OR scores OR index OR equation* OR formul* OR tool*)) OR nomogram* OR ((neural) NEAR/3 (network*))):ab,ti OR predict*:ti))

((metasta* OR second-canc* OR radioligand*):ti)

("conference abstract":kw OR Trial registry record:pt)

#1 NOT #2 NOT #3

**Web of Science**

TS=(((prostat*) NEAR/2 (tumor* OR tumour* OR neoplas* OR carcino* OR cancer* OR malignan* OR adenoma* OR adenocarcinoma* OR radiotherap* OR brachytherap*))) **AND** TS=(radiotherap* OR brachytherap* OR radiation* OR irradiat* OR ((radio* OR irradiat* OR proton* OR arc) NEAR/2 (therap* OR treatment*)) OR EBRT OR iodine-125* OR iodide-125* OR I125 OR 125I) **AND** (TS=(((predict* OR forecast* OR prognos*) NEAR/2 (model* OR score OR scores OR index OR equation* OR formul* OR tool*)) OR nomogram* OR ((neural) NEAR/2 (network*))) OR TI=predict*) NOT TI=(metasta* OR second-canc* OR radioligand*) NOT TS=((animal* OR rat OR rats OR mouse OR mice OR murine OR dog OR dogs OR canine OR cat OR cats OR feline OR rabbit OR cow OR cows OR bovine OR rodent* OR sheep OR ovine OR pig OR swine OR porcine OR veterinar* OR chick* OR zebrafish* OR baboon* OR nonhuman* OR primate* OR cattle* OR goose OR geese OR duck OR macaque* OR avian* OR bird* OR fish*) NOT (human* OR patient* OR women OR woman OR men OR man)) NOT DT=(Meeting Abstract OR Meeting Summary) NOT DT=(Editorial Material)

**Google Scholar**

"prostate|prostatic tumor|tumour|neoplasm|carcinoma|cancer|malignancy" radiotherapy|brachytherapy|radiation|irradiation|"radio|proton|arc therapy|treatment"|EBRT prediction|predictive -metastasis -radioligand -animal -mouse -mice -rat

'prostate|prostatic tumor|tumour|neoplasm|carcinoma|cancer|malignancy' radiotherapy|brachytherapy|radiation|irradiation|'radio|proton|arc therapy|treatment'|EBRT prediction|predictive -metastasis -radioligand -animal -mouse -mice -rat

## 2. TRIPOD SRMA-checklist for reporting systematic reviews of prediction model studies

| **Section and**  **topic** | **Item**  **No** | **Checklist item** | **Page** |
| --- | --- | --- | --- |
| **Title** |  |  |  |
| Title | 1 | Identify the report as a systematic review or meta-analysis (or both) of diagnostic or prognostic model studies. Specify the target population and  outcome(s) predicted as relevant to the review question. | 1 |
| **Abstract** |  |  |  |
| Abstract | 2 | See the TRIPOD-SRMA Checklist for Abstracts | 1 |
| **Introduction** |  |  |  |
| Rationale | 3 | Describe the rationale for the review in the context of existing knowledge. | 2 |
| Objectives | 4 | Provide an explicit statement of the objective(s) being addressed with reference to: target population, index and comparator models (as relevant),  outcome(s), time (prediction horizon and intended moment of using the model), and setting. | 2 |
| **Methods** |  |  |  |
| Study eligibility  criteria | 5 | Specify study characteristics used as eligibility criteria, including any prediction models of specific interest, and whether development or validation  studies (or both) were eligible. | 3-4 |
| Information  sources | 6 | Specify all databases, registers, websites, organisations, reference lists and other sources searched or consulted to identify studies. Specify the date  when each source was last searched or consulted. | 3 |
| Search strategy | 7 | Present the full search strategies for all databases, registers and websites, including any filters and limits used. | Supplementary A |
| Study selection  process | 8 | Specify the methods used to decide whether a study met the inclusion criteria of the review, including how many reviewers screened each record  and each report retrieved, whether they worked independently, and if applicable, details of automation tools used in the process. | 4-5 |
| Data collection process | 9 | Specify the methods used to collect data from study reports, including how many reviewers collected data from each report, whether they worked independently, any processes for obtaining or confirming data from study investigators, and if applicable, details of automat ion tools used in the  process. | 5 |
| Data Items | 10a | List and define all items for which data were sought from each study. | 5 |
|  | 10b | State the model performance measures that were sought (e.g., measures of calibration, discrimination, overall model fit, clinical utility). | 5 |
|  | 10c | Describe how any desired but unreported data items (items 10a, 10b) were handled (e.g., contacted authors, calculated from other reported  information). | Authors were not contacted since the study aimed to capture limitations in prediction modeling reporting |
| Risk of bias and applicability  assessment | 11 | Specify the methods used to assess risk of bias in the included studies and their applicability to the review question. This should be done separately for each model development and validation. Include details of any tool(s) used, how many reviewers assessed each study and whether they worked  independently. | 5-6 |
| Synthesis methods | 12a | Describe any methods for synthesising estimates of performance measures for each model. If meta-analysis was carried out, describe the methods used, including any transformations of data prior to pooling, how any heterogeneity in model performance was quantified and handled, and  software package(s) used. | 5 |
|  | 12b | Describe any methods used to explore possible causes of heterogeneity in model performance (e.g., subgroup analysis, meta-regression), including  whether or not they were planned. | Not performed |

|  | 12c | Describe any sensitivity analyses conducted to assess robustness of the synthesised results. | Not performed |
| --- | --- | --- | --- |
| Certainty  assessment | 13 | Describe any methods used to assess certainty (or confidence) in the body of evidence for a prediction model. | Risk of bias assessment |
| **Results** |  |  |  |
| Study selection | 14 | Describe the results of the search and selection process, from the number of records identified in the search to the number of studies and models included in the review, ideally using a flow diagram. | 6 |
| Study and model  characteristics | 15 | Present study characteristics and model details extracted (as per Item 10a), and cite the study reports. | Supplementary B |
| Risk of bias and  applicability | 16 | Present results of risk of bias and applicability assessment. This should be done separately for each model development and validation in each  included study. | Supplementary C |
| Results of model performance in individual  studies | 17 | Present performance estimates and confidence intervals for each model and all evaluations, including whether they relate to the internal or external validation performance. If internal, give details of the method. | Supplementary B |
| Results of syntheses | 18a | Present the results of any synthesis of model performance, together with details of which study estimates contributed. If meta-analysis was carried  out, then for each model and performance measure, present summary results, confidence/credible intervals and measures of heterogeneity. Forest plots may be useful. | 9-10 |
|  | 18b | For each model, present results of all investigations of possible causes of heterogeneity in model performance. | Not performed |
|  | 18c | Present results of all sensitivity analyses conducted to assess the robustness of the synthesised results. | Not performed |
| Certainty of  evidence | 19 | Present any assessments of certainty (or confidence) in the body of evidence for each prediction model of interest. | Supplementary B |
| **Discussion** |  |  |  |
| Summary of  evidence | 20 | Summarise the main findings including the strengths and limitations of the evidence. | 12-14 |
| Limitations | 21 | Discuss the strengths and limitations of the review process. | 14 |
| Implications | 22 | Discuss implications of the results in the context of other evidence and for practice, policy, and future research. | 14-15 |
| **Other**  **information** |  |  |  |
| Registration and protocol | 23a | Provide registration information for the review, including register name and registration number, or state that the review was not registered. | 3 |
|  | 23b | Indicate where the review protocol can be accessed, or state that a protocol was not prepared. | 3 |
|  | 23c | Describe and explain any amendments to information provided at registration or in the protocol. | - |
| Support | 24 | Describe sources of financial or non-financial support for the review, and the role of the funders or sponsors in the review. | 15-16 |
| Competing  interests | 25 | Declare any competing interests of review authors. | 15 |

| Availability of  data, code, and other materials | 26 | Report which of the following are publicly available and where they can be found: template data collection forms; data extrac ted from included studies; data used for all analyses; analytic code; any other materials used in the review. | Supplementary material |
| --- | --- | --- | --- |

This checklist appears in appendix 2 of Snell KIE, Levis B, Damen JAA, et al. Transparent reporting of multivariable prediction models for individual prognosis or diagnosis: checklist for systematic reviews and meta-analyses (TRIPOD-SRMA). *BMJ* 2023;381:e073538. doi:10.1136/bmj-2022-073538.

## 3. Figure displaying outcome distribution of the included studies

*Figure 1 Distribution of 107 models from the 34 included studies per outcome and time-horizon.*
*Note: Overall incidence refers to outcomes with an undefined onset and a late resolution time.*

## 4. Supplementary tables

*Table 1 Predictor importance for genitourinary outcomes*

|  |  | **Overall GU toxicity** | | **Urinary incontinence** | | **Hemaeturia** | | **Dysuria** | | **Urinary frequency** | |
| --- | --- | --- | --- | --- | --- | --- | --- | --- | --- | --- | --- |
| **Predictors** | | *Nr times included as candidate* | *Frequency of selection in final models* | *Nr times included as candidate* | *Frequency of selection in final models* | *Nr times included as candidate* | *Frequency of selection in final models* | *Nr times included as candidate* | *Frequency of selection in final models* | *Nr times included as candidate* | *Frequency of selection in final models* |
|  | Baseline score/symptoms | 14 | 71% | 179 | 24% | 5 | 20% | 5 | 100% | 6 | 33% |
| Patient characteristics | Age | 8 | 38% | 24 | 38% | 6 | 17% | 6 | 33% | 6 | 17% |
|  | BMI | 7 | 29% | 15 | 27% | 4 | 25% | 5 | 60% | 6 | 17% |
|  | Smoking | 15 | 7% | 15 | 0% | 6 | 0% | 6 | 33% | 6 | 33% |
|  | ECOG | 0 | #DIV/0! | 5 | 20% | 4 | 0% | 5 | 20% | 5 | 60% |
|  | Prostate volume | 1 | 100% | 3 | 33% | 0 | #DIV/0! | 0 | #DIV/0! | 0 | #DIV/0! |
|  | Alcohol intake | 6 | 0% | 11 | 0% | 4 | 0% | 5 | 0% | 6 | 0% |
| Comorbidities | Diabetes Mellitus | 4 | 25% | 26 | 8% | 9 | 11% | 10 | 40% | 11 | 0% |
|  | Cardiovascular diseases | 7 | 29% | 31 | 13% | 14 | 7% | 16 | 6% | 16 | 0% |
|  | Gastrointestinal disorders | 2 | 0% | 8 | 38% | 5 | 40% | 5 | 40% | 6 | 0% |
|  | Cerebrovascular disorders | 0 | #DIV/0! | 5 | 40% | 4 | 0% | 5 | 60% | 5 | 20% |
|  | Chronic Diseases | 1 | 0% | 6 | 0% | 0 | #DIV/0! | 0 | #DIV/0! | 0 | #DIV/0! |
| Medical history | TURP | 7 | 0% | 9 | 67% | 2 | 0% | 1 | 100% | 1 | 0% |
|  | Previous surgery | 6 | 0% | 10 | 30% | 2 | 0% | 1 | 0% | 1 | 0% |
| Medication-intake | Antihypertensives | 11 | 73% | 11 | 9% | 4 | 0% | 5 | 20% | 6 | 17% |
|  | Cardiovascular drugs | 6 | 100% | 6 | 0% | 0 | #DIV/0! | 0 | #DIV/0! | 1 | 0% |
|  | Antihypercholesterolemic drugs | 7 | 29% | 5 | 0% | 4 | 25% | 5 | 40% | 6 | 0% |
|  | Anticoagulants | 4 | 0% | 17 | 6% | 5 | 0% | 6 | 17% | 7 | 14% |
|  | NSAID | 0 | #DIV/0! | 5 | 0% | 4 | 0% | 5 | 20% | 5 | 40% |
|  | 5-alpha-reductase inhibitors | 6 | 33% | 0 | #DIV/0! | 0 | #DIV/0! | 0 | #DIV/0! | 1 | 0% |
|  | Antidepressives | 2 | 0% | 6 | 0% | 0 | #DIV/0! | 0 | #DIV/0! | 1 | 0% |
|  | Hypercholesterolemia | 0 | #DIV/0! | 11 | 0% | 4 | 0% | 5 | 0% | 5 | 0% |
| Tumor characteristics | Clinical Staging (T or N) | 11 | 73% | 24 | 25% | 3 | 0% | 3 | 0% | 1 | 0% |
|  | PSA or Gleason grade | 2 | 0% | 35 | 9% | 5 | 0% | 2 | 0% | 0 | #DIV/0! |
| Treatment-related predictors | ADT | 7 | 57% | 25 | 8% | 2 | 0% | 1 | 0% | 1 | 0% |
|  | RT dose | 3 | 0% | 5 | 20% | 4 | 0% | 5 | 20% | 6 | 33% |
|  | RT type | 0 | #DIV/0! | 9 | 67% | 0 | #DIV/0! | 0 | #DIV/0! | 0 | #DIV/0! |
|  | Extended RT | 20 | 0% | 12 | 0% | 1 | 0% | 0 | #DIV/0! | 4 | 0% |
|  | Fractionation | 2 | 0% | 0 | #DIV/0! | 0 | #DIV/0! | 0 | #DIV/0! | 1 | 0% |

*Abbreviations: ADT (Androgen deprivation therapy); BMI (Body Mass Index) ; Eastern Cooperative Oncology Group Performance Status (ECOG); Nr (Number); NSAID (Non-steroidal anti-inflammatory drugs); RT (Radiotherapy)*

*Table 2 Predictor importance for gastrointestinal outcomes*

|  |  | **Overall GI toxicity** | | **Fecal incontinence** | | **Rectal bleeding** | |
| --- | --- | --- | --- | --- | --- | --- | --- |
| **Predictors** | | *Nr times included as candidate* | *Frequency of selection in final models* | *Nr times included as candidate* | *Frequency of selection in final models* | *Nr times included as candidate* | *Frequency of selection in final models* |
|  | Baseline score/ symptoms | 3 | 100% | 8 | 13% | 4 | 0% |
|  | Acute toxicity | 1 | 100% | 0 | #DIV/0! | 2 | 50% |
| Patient characteristics | Age | 8 | 25% | 5 | 100% | 7 | 0% |
|  | Hemorrhoids | 4 | 25% | 2 | 50% | 3 | 67% |
|  | Platelet Count | 0 | #DIV/0! | 0 | #DIV/0! | 1 | 100% |
|  | Smoking | 4 | 0% | 1 | 0% | 0 | #DIV/0! |
|  | Prostate volume | 4 | 0% | 4 | 0% | 4 | 0% |
|  | BMI | 3 | 0% | 1 | 0% | 0 | #DIV/0! |
| Comorbidities | Gastrointestinal disorders | 0 | #DIV/0! | 4 | 0% | 4 | 0% |
|  | Diabetes Mellitus | 9 | 33% | 2 | 0% | 2 | 0% |
|  | Cardiovascular diseases | 6 | 0% | 2 | 0% | 4 | 25% |
|  | Nr chronic diseases | 3 | 0% | 0 | #DIV/0! | 0 | #DIV/0! |
|  | Autoimmune diseases | 3 | 0% | 1 | 0% | 0 | #DIV/0! |
| Medical history | Previous surgery | 1 | 0% | 5 | 20% | 7 | 29% |
|  | TURP | 0 | #DIV/0! | 4 | 0% | 4 | 0% |
| Medication-intake | Antihypercholesterolemic Drugs | 4 | 100% | 0 | #DIV/0! | 0 | #DIV/0! |
|  | Anticoagulants | 1 | 100% | 1 | 0% | 4 | 50% |
|  | Antihypertensives | 2 | 50% | 2 | 50% | 3 | 33% |
|  | Metformin | 4 | 0% | 0 | #DIV/0! | 0 | #DIV/0! |
|  | Alpha-blockers | 4 | 0% | 0 | #DIV/0! | 0 | #DIV/0! |
| Tumor characteristics | PSA or Gleason grade | 17 | 24% | 8 | 13% | 9 | 44% |
|  | Risk group | 3 | 0% | 0 | #DIV/0! | 2 | 0% |
|  | Clinical Staging | 6 | 0% | 0 | #DIV/0! | 1 | 0% |
| **Other predictors** | |  |  |  |  |  |  |
| Treatment-related factors | ADT | 10 | 10% | 7 | 0% | 9 | 11% |
|  | RT type | 3 | 33% | 6 | 0% | 5 | 0% |
|  | RT with irradiation to pelvic  nodes or seminal vesicles | 14 | 7% | 2 | 0% | 2 | 0% |
|  | RT dose | 3 | 0% | 0 | #DIV/0! | 1 | 0% |

*Abbreviations: ADT (Androgen deprivation therapy); BMI (Body Mass Index); Nr (Number); NSAID (Non-steroidal anti-inflammatory drugs); RT (Radiotherapy)*

*Table 3 Risk of bias assessment of the best performing models (AUC ≥ 0.70) with an acceptable methodological quality (at least four out of six SF-PROBAST criteria met)*

| *Authors development* | *Outcome* | *Calibration slope development* | *Calibration slope validation* | *Discriminative ability Development* | *Discriminative ability Validation* | *Outcome assessment (Y/N)* | *Events per variable (Y/N/NI)* | *Continuous predictors (Y/N/NI)* | *Missing data (Y/N)* | *Univariate analysis (Y/N/U)* | *Correction for overfitting/optimism (Y/N)* | *Final score  (SF-PROBAST)* |
| --- | --- | --- | --- | --- | --- | --- | --- | --- | --- | --- | --- | --- |
| *David et al* (1) | *G2+ urinary toxicity* | *NR* | *NA* | *0,80* | *NA* | *Y* | *Y* | *Y* | *Y* | *Y* | *Y* | *0* |
| *Huang et al* (2) | *G2+ rectal bleeding* | *NR* | *NA* | *0,87* | *NA* | *Y* | *N* | *Y* | *N* | *Y* | *Y* | *2* |
| *Rossi et al* (3) | *Laser treatment or transfusion required after rectal bleeding* | *NR* | *NA* | *0,73* | *NA* | *Y* | *N* | *Y* | *Y* | *N* | *Y* | *2* |
| *Rossi et al* (3) | *Laser treatment or transfusion required after rectal bleeding* | *NR* | *NA* | *0,72* | *NA* | *Y* | *N* | *Y* | *Y* | *N* | *Y* | *2* |
| *Rossi et al* (3) | *Use of diapers due to fecal incontinence* | *NR* | *NA* | *0,73* | *NA* | *Y* | *N* | *Y* | *Y* | *N* | *Y* | *2* |
| *Rossi et al* (3) | *Use of diapers due to fecal incontinence* | *NR* | *NA* | *0,75* | *NA* | *Y* | *N* | *Y* | *Y* | *N* | *Y* | *2* |
| *Rossi et al* (3) | *Use of diapers due to urinary incontinence* | *NR* | *NA* | *0,71* | *NA* | *Y* | *N* | *Y* | *Y* | *N* | *Y* | *2* |
| *Rossi et al* (3) | *Use of diapers due to urinary incontinence* | *NR* | *NA* | *0,71* | *NA* | *Y* | *N* | *Y* | *Y* | *N* | *Y* | *2* |
| *Rossi et al* (3) | *Use of diapers due to urinary incontinence* | *NR* | *NA* | *0,73* | *NA* | *Y* | *N* | *Y* | *Y* | *N* | *Y* | *2* |
| *Sini et al* (4) | *Loose stools: ΔIBDQ5 < -2 (maximum variation)* | *1,00* | *NA* | *0,71* | *NA* | *Y* | *N* | *Y* | *Y* | *N* | *Y* | *2* |
| *Carillo et al* (5) | *IPSS ≥ 15* | *1,10* | *NA* | *0,71* | *NA* | *Y* | *N* | *Y* | *Y* | *N* | *Y* | *2* |
| *Carillo et al* (5) | *IPSS ≥ 15* | *NR* | *NA* | *0,76* | *NA* | *Y* | *N* | *Y* | *Y* | *N* | *Y* | *2* |
| *Carillo et al* (5) | *IPSS ≥ 15* | *NR* | *NA* | *0,75* | *NA* | *Y* | *N* | *Y* | *Y* | *N* | *Y* | *2* |
| *Carillo et al* (5) | *IPSS ≥ 15* | *NR* | *NA* | *0,76* | *NA* | *Y* | *N* | *Y* | *Y* | *N* | *Y* | *2* |
| *Carillo et al* (5) | *IPSS ≥ 15* | *NR* | *NA* | *0,76* | *NA* | *Y* | *N* | *Y* | *Y* | *N* | *Y* | *2* |
| *Carillo et al* (5) | *IPSS ≥ 15* | *NR* | *NA* | *0,73* | *NA* | *Y* | *N* | *Y* | *Y* | *N* | *Y* | *2* |
| *Carillo et al* (5) | *IPSS ≥ 15* | *NR* | *NA* | *0,73* | *NA* | *Y* | *N* | *Y* | *Y* | *N* | *Y* | *2* |
| *Carillo et al* (5) | *IPSS ≥ 15* | *NR* | *NA* | *0,75* | *NA* | *Y* | *N* | *Y* | *Y* | *N* | *Y* | *2* |
| *Palorini et al* (6) | *IPSS ≥ 15* | *1,03* | *NR* | *0,71* | *NR* | *Y* | *N* | *Y* | *N* | *Y* | *Y* | *2* |
| *Yahya et al* (7) | *G1+ Dysuria* | *0,60* | *NA* | *0,78* | *NA* | *Y* | *N* | *Y* | *Y* | *Y* | *Y* | *1* |
| *Yahya et al* (7) | *G1+ Hematuria* | *0,68* | *NA* | *0,79* | *NA* | *Y* | *N* | *Y* | *Y* | *Y* | *Y* | *1* |
| *Hasannejadasl et al* (9) | *Erectile dysfunction* | *NR* | *NR* | *NR* | *0,84* | *Y* | *N* | *Y* | *N* | *Y* | *Y* | *2* |
| *Hasannejadasl et al* (9) | *Erectile dysfunction* | *NR* | *NR* | *NR* | *0,81* | *Y* | *N* | *Y* | *N* | *Y* | *Y* | *2* |
| *Hasannejadasl et al* (8) | *Urinary incontinence* | *NR* | *NR* | *NR* | *0,79* | *Y* | *N* | *N* | *Y* | *Y* | *Y* | *2* |
| *Hasannejadasl et al* (8) | *Urinary incontinence* | *NR* | *NR* | *NR* | *0,74* | *Y* | *N* | *N* | *Y* | *Y* | *Y* | *2* |
| *Hasannejadasl et al* (8) | *Urinary incontinence* | *NR* | *NR* | *NR* | *0,71* | *Y* | *N* | *N* | *Y* | *Y* | *Y* | *2* |
| *Hasannejadasl et al* (8) | *Urinary incontinence* | *NR* | *NR* | *NR* | *0,76* | *Y* | *N* | *N* | *Y* | *Y* | *Y* | *2* |
| *Hasannejadasl et al* (8) | *Urinary incontinence* | *NR* | *NR* | *NR* | *0,72* | *Y* | *N* | *N* | *Y* | *Y* | *Y* | *2* |
| *Cozzarini et al* (10) | *Nocturia  (IPSS ≥ 4)* | *NR* | *0,66* | *0,75* | *0,70* | *Y* | *N* | *Y* | *Y* | *Y* | *Y* | *1* |
| *Roeloffzen et al* (11) | *Urinary retention requiring catheterization* | *NR* | *NR* | *0,82* | *0,86* | *Y* | *N* | *Y* | *Y* | *Y* | *Y* | *1* |
| *Alemozaffar et al* (12) | *Erection suitable for intercourse* | *NR* | *NR* | *0,83* | *0,87* | *Y* | *N* | *N* | *Y* | *Y* | *Y* | *2* |
| *Alemozaffar et al* (12) | *Erection suitable for intercourse* | *NR* | *NR* | *0,89* | *0,90* | *Y* | *N* | *NI* | *Y* | *Y* | *Y* | *2* |

Note: the final score (SF-PROBAST score) refers to how many criteria were not met.
Abbreviations: *IPSS (International Prostate Symptom Score);* N (No); NA (Not applicable – external validation not performed); NI (No information); NR (Not reported); Y (Yes)

## 5. Risk of bias assessment with the short -form (including explanation)

**Short form guidelines** (13)

This short form consists of 6 PROBAST questions that can be answered with “yes”, “no”, or “no information”. Each incorrectly performed item (ie, answered with “no”) counts as 1 point, allowing a total score of 0 to 6 points. Models are categorized based on the total score, with a score of 1 or more indicating high ROB. Models with 0 points are rated low ROB or, when at least 1 item was answered with “no information”, unclear ROB.

The effect of using univariable selection and not correcting for optimism will be negligible when the effective sample size is very large. Therefore, the points for these items will be dropped when the EPV is very large (definition: EPV candidate predictors ≥25, or, when the number of candidate predictors is missing, EPV final model ≥50).

Important changes or additions to the definitions in the original PROBAST tool are marked red.

1) **Outcome assessment** – combination of PROBAST questions 3.1, 3.2, and 3.4

*Was the outcome determined appropriately in a similar way for all patients, using a standard measure or definition?*

a. **Yes:**
   1. A method of outcome determination has been used which is considered optimal or acceptable by guidelines or previous publications on the topic.

Note: This is about level of measurement error within the method of determining outcome, not about whether the definition of the outcome method is appropriate.

2. *And*, the method of outcome determination is objective *or* a standard outcome definition is used *or* pre-specified categories are used to group outcomes.
3. *And*, outcomes were defined and determined in a similar way for all participants.
b. **No:**
   1. A clearly suboptimal method has been used that causes unacceptable error in determining outcome status in participants or if a measure that involves subjective judgment or special training was used by inexperienced persons.
   2. *Or*, the outcome definition was not standard and not pre-specified.
   3. *Or*, outcomes were clearly defined and determined in a different way for some participants.

Notes & examples:

Mortality is an example of a very objective measure and will often be rated as “yes”, when determined in a similar way for all patients.

Using the modified Rankin Scale (a score ranging from 0 to 6) is considered to be an accepted outcome measure for stroke, but can still be rated as “no” when assessed by inexperienced trainees.

2) **Events per variable (EPV)** – PROBAST question 4.1

*Were there a reasonable number of participants with the outcome?*

a. **Yes:** the ratio of the number of participants with the outcome relative to the number of candidate predictors is at least 20, or relative to the number of final covariates at least 40 (EPV candidates ≥20 or EPV final model ≥40).
b. **No:** the ratio of the number of participants with the outcome relative to the number of candidate predictors is less than 10, or relative to the number of final covariates less than 20 (EPV candidates <10 or EPV final model <20).
c. **No information:**
   1. There is no information on the number of (candidate) predictor parameters or number of participants with the outcome, such that the EPV cannot be calculated.
   2. *Or*, the ratio of the number of participants with the outcome relative to the number of candidate predictors is between 10 and 20, or relative to the number of final covariates between 20 and 40 (EPV candidates 10-20 or EPV final model 20-40).

Notes & examples:

Only when the number of candidate predictors is unclear, the number of items reported in the univariable analysis should be used. If that is not reported, than the number of parameters in the final model is used.

Candidate variables = all variables that were used in the univariable or multivariable selection process. To calculate the EPV, use the number of candidate predictor parameters: continuous variable = 1 parameter, non-linear variable (eg, exponential, fractional polynomial or spline) = 2 parameters, categorical variable = number of categories minus 1.

Examples of the number of candidate predictors:

1. Age (continuous) = 1 parameter
2. Gender (male/female) = 1 parameter
3. Blood pressure (odds ratio for every mmHg <120 & odds ratio for every mmHg >120) = non-linear = 2 parameters
4. Location of occlusion (M1/M2/ICA/other) = 4 categories = 3 parameters
3) **Continuous predictors** – PROBAST question 4.2

*Were continuous and categorical predictors handled appropriately?*

a. **Yes:**
   1. Continuous predictors are not converted into ≥2 categories (ie, dichotomized or categorized) when included in the model.
   2. *Or,* continuous predictors are converted into ≥2 categories based on pre-specified or widely accepted cut points (this should be explicitly mentioned in the paper or statistical protocol).
   3. *Or,* continuous predictors are examined for non-linearity (eg, with exponential, fractional polynomial or spline function).
   4. *Or,* categorical predictor groups are defined using a pre-specified or widely accepted method (ie, not based on the own data).
b. **No:** continuous predictors are converted into ≥2 categories *or* categorical predictor groups definitions are based on the “optimal” cut point or threshold in the current dataset.
c. **No information:**
   1. Continuous predictors are converted into ≥2 categories but it is not mentioned how these cut points were chosen.
   2. *Or*, it is unclear how categorical predictor groups were defined.

Notes & examples:

Dichotomizing means that a variable with continuous values is split in 2 or more groups (eg, age <65 and age ≥65), which causes loss of efficiency. Is can also cause risk of bias when the cut points are based on the current dataset.

Pre-specified = cut points or categories that were determined in the papers methods section or in a previously published paper.

If the cut points themselves are not specified but it is specified how to get the cut point (eg, use the median), it can be rated “yes” (it may not be good modeling practice, but it should not lead to high risk of bias).

4) **Missing data** – PROBAST question 4.4

*Were participants with missing data handled appropriately?*

a. **Yes:**
   1. The study explicitly reports that there are no missing values of predictors or outcomes.
   2. *Or*, missing values are handled using multiple imputation.
   3. *Or,* <5% of all patients were excluded based on missing values.
   4. *Or,* a clear justification was declared for the method of missing data handling.
b. **No:**
   1. >5% or an unknown percentage of participants with missing data are omitted from the analysis.
   2. *Or*, the method of handling missing data is clearly flawed (eg, single imputation, missing indicator method or inappropriate use of last value carried forward).
   3. *Or,* the study had no explicit mention of methods to handle missing data.

Notes & examples:

If there is no explicit mention missing data or the methods to handle missing data, this question should be answered as “no” and not as “no information”.

Adding missing values to the reference class of a categorical variable can only be rated appropriate (ie, “yes”) when at least 80% of the patients are in the reference class and <10% of the values are missing.

Using a separate category for missing values of a categorical variable is only appropriate when the model is applied to a similar setting (ie, when the pattern of missingness and the cause of missing values in the validation cohort will be similar as in the derivation cohort), but in general it should be rated as “no”.

5) **Univariable analysis** – PROBAST question 4.5

No points will be counted for this item when the EPV is very large (definition: EPV candidate predictors ≥25, or, when the number of candidate predictors is missing, EPV final model ≥50).

*Was selection of predictor based on univariable analysis avoided?*

a. **Yes:** the predictors are *not* selected based on univariable analysis prior to multivariable modeling (ie, predictors in the multivariable analysis were pre-specified or chosen based on expert opinion).
b. **No:** the predictors are selected based on univariable analysis prior to multivariable modelling (ie, predictors were first analyzed one at a time and only included in the multivariable model if this analysis was significant).
c. **Unclear:** there is insufficient information to indicate that univariable selection is avoided.

Notes & examples:

If there is no evidence of univariable selection, the question should be answered “yes” (ie, the authors don’t have to mention specifically that they didn’t use it).

If univariable analysis was performed, but the authors explicitly mention that predictors were included in the multivariable analysis regardless of these results, it should be rated “yes”.

If univariable analysis was performed, but it is not clear whether this affected predictor selection for the multivariable model, it should be rated “no information”.

Exception: if it is explicitly mentioned that the internal validation process included all variable selection procedures, this question can be answered with “yes”.

6) **Correction for overfitting/optimism** – PROBAST question 4.8

No points will be counted for this item when the EPV is very large (definition: EPV candidate predictors ≥25, or, when the number of candidate predictors is missing, EPV final model ≥50).

*Was model overfitting and optimism in model performance accounted for?*

a. **Yes:** internal validation techniques, such as bootstrapping and cross-validation, have been used to account for any optimism in model fitting, *and* subsequent adjustment of the prediction model performance measures have been applied (ie, “internally validated AUC”).
b. **No:**
   1. No internal validation has been performed.
   2. *Or*, internal validation consisted only of a single random split-sample of participant data.
   3. *Or,* the bootstrapping or cross-validation did not include all model development procedures including any variable selection.

Notes & examples

Internal validation and adjustment of model performance measures can be indicated in the text with for example: “bootstrapping”, “optimism correction”, “lasso”, “penalized regression”, or “shrinkage”. A random split sample is not considered a valid internal validation technique.

If there is nothing reported about internal validation or correction for overfitting/optimism, we assume that it has not been performed (so this item should be rated “no”).

External validation is not integrated in the PROBAST short form. Only internal validation is used to correct the apparent performance measure in the development cohort (ie, derivation AUC) for optimism, and can thereby reduce bias of the reported AUC.

## 6. References

1.	David R, Hiwase M, Kahokehr AA, Lee J, Watson DI, Leung J, e.a. Predicting post-radiation genitourinary hospital admissions in patients with localised prostate cancer. World J Urol. 2022;40(12):2911-8. doi:10.1007/s00345-022-04212-y PubMed PMID: 36357601; PubMed Central PMCID: PMC9712379.

2.	Huang CC, Chao PJ, Guo SS, Wang CJ, Luo HL, Su YL, e.a. Developing a multivariable normal tissue complication probability model to predict late rectal bleeding following intensity-modulated radiation therapy. J Cancer. 2019;10(11):2588-93. doi:10.7150/jca.29606 PubMed PMID: 31258765; PubMed Central PMCID: PMC6584341.

3.	Rossi L, Bijman R, Schillemans W, Aluwini S, Cavedon C, Witte M, e.a. Texture analysis of 3D dose distributions for predictive modelling of toxicity rates in radiotherapy. Radiother Oncol J Eur Soc Ther Radiol Oncol. december 2018;129(3):548-53. doi:10.1016/j.radonc.2018.07.027 PubMed PMID: 30177372.

4.	Sini C, Noris Chiorda B, Gabriele P, Sanguineti G, Morlino S, Badenchini F, e.a. Patient-reported intestinal toxicity from whole pelvis intensity-modulated radiotherapy: First quantification of bowel dose-volume effects. Radiother Oncol J Eur Soc Ther Radiol Oncol. augustus 2017;124(2):296-301. doi:10.1016/j.radonc.2017.07.005 PubMed PMID: 28739383.

5.	Carillo V, Cozzarini C, Rancati T, Avuzzi B, Botti A, Borca VC, e.a. Relationships between bladder dose-volume/surface histograms and acute urinary toxicity after radiotherapy for prostate cancer. Radiother Oncol J Eur Soc Ther Radiol Oncol. april 2014;111(1):100-5. doi:10.1016/j.radonc.2014.02.006 PubMed PMID: 24631144.

6.	Palorini F, Rancati T, Cozzarini C, Improta I, Carillo V, Avuzzi B, e.a. Multi-variable models of large International Prostate Symptom Score worsening at the end of therapy in prostate cancer radiotherapy. Radiother Oncol J Eur Soc Ther Radiol Oncol. januari 2016;118(1):92-8. doi:10.1016/j.radonc.2015.11.036 PubMed PMID: 26777123.

7.	Yahya N, Ebert MA, Bulsara M, Haworth A, Kennedy A, Joseph DJ, e.a. Dosimetry, clinical factors and medication intake influencing urinary symptoms after prostate radiotherapy: An analysis of data from the RADAR prostate radiotherapy trial. Radiother Oncol J Eur Soc Ther Radiol Oncol. juli 2015;116(1):112-8. doi:10.1016/j.radonc.2015.06.011 PubMed PMID: 26163088.

8.	Hasannejadasl H, Osong B, Bermejo I, van der Poel H, Vanneste B, van Roermund J, e.a. A comparison of machine learning models for predicting urinary incontinence in men with localized prostate cancer. Front Oncol. 2023;13:1168219. doi:10.3389/fonc.2023.1168219 PubMed PMID: 37124522; PubMed Central PMCID: PMC10130634.

9.	Hasannejadasl H, Roumen C, van der Poel H, Vanneste B, van Roermund J, Aben K, e.a. Development and external validation of multivariate prediction models for erectile dysfunction in men with localized prostate cancer. PLOS ONE. 3 maart 2023;18(3):e0276815. doi:10.1371/journal.pone.0276815 PubMed PMID: 36867616; PubMed Central PMCID: PMC9983834.

10.	Cozzarini C, Rancati T, Carillo V, Civardi F, Garibaldi E, Franco P, e.a. Multi-variable models predicting specific patient-reported acute urinary symptoms after radiotherapy for prostate cancer: Results of a cohort study. Radiother Oncol J Eur Soc Ther Radiol Oncol. augustus 2015;116(2):185-91. doi:10.1016/j.radonc.2015.07.048 PubMed PMID: 26276529.

11.	Roeloffzen EM, van Vulpen M, Battermann JJ, van Roermund JG, Saibishkumar EP, Monninkhof EM. Pretreatment nomogram to predict the risk of acute urinary retention after I-125 prostate brachytherapy. Int J Radiat Oncol Biol Phys. 1 november 2011;81(3):737-44. doi:10.1016/j.ijrobp.2010.06.009 PubMed PMID: 20888131.

12.	Alemozaffar M, Regan MM, Cooperberg MR, Wei JT, Michalski JM, Sandler HM, e.a. Prediction of erectile function following treatment for prostate cancer. JAMA. 21 september 2011;306(11):1205-14. doi:10.1001/jama.2011.1333 PubMed PMID: 21934053; PubMed Central PMCID: PMC3831607.

13.	Venema E, Wessler BS, Paulus JK, Salah R, Raman G, Leung LY, e.a. Large-scale validation of the prediction model risk of bias assessment Tool (PROBAST) using a short form: high risk of bias models show poorer discrimination. J Clin Epidemiol. oktober 2021;138:32-9. doi:10.1016/j.jclinepi.2021.06.017 PubMed PMID: 34175377.
